# Application of information theoretic feature selection and machine learning methods for the development of genetic risk prediction models

**DOI:** 10.1038/s41598-021-00854-x

**Published:** 2021-12-02

**Authors:** Farideh Jalali-najafabadi, Michael Stadler, Nick Dand, Deepak Jadon, Mehreen Soomro, Pauline Ho, Helen Marzo-Ortega, Philip Helliwell, Eleanor Korendowych, Michael A. Simpson, Jonathan Packham, Catherine H. Smith, Jonathan N. Barker, Neil McHugh, Richard B. Warren, Anne Barton, John Bowes, BADBIR Study Group, Catherine H. Smith, Jonathan N. Barker, Richard B. Warren, BSTOP Study Group, Nick Dand, Catherine H. Smith

**Affiliations:** 1 Centre for Genetics and Genomics Versus Arthritis,Centre for Musculoskeletal Research,Faculty of Biology, Medicine and Health, Manchester Academic Health Science Centre, The University of Manchester, Manchester, M13 9PT UK; 2 Department of Medical and Molecular Genetics, Faculty of Life Sciences and Medicine, King’s College London, London , UK; 3 Department of Medicine, University of Cambridge, Cambridge, UK; 4 NIHR Manchester Musculoskeletal Biomedical Research Unit,Central Manchester NHS Foundation Trust, Manchester Academic Health Science Centre, Manchester, UK; 5 NIHR Leeds Biomedical Research Centre, Leeds Teaching Hospitals Trust and Leeds Institute of Rheumatic and Musculoskeletal Disease, University of Leeds, Manchester, UK; 6 Royal National Hospital for Rheumatic Diseases and Dept Pharmacy and Pharmacology, University of Bath, Bath , UK; 7 Division of Epidemiology and Public Health, University of Nottingham, Nottingham , UK; 8 St John’s Institute of Dermatology, Guys and St Thomas’ Foundation Trust, London, UK; 9 St John’s Institute of Dermatology, Faculty of Life Sciences and Medicine, King’s College London, London, UK; 10 Dermatology Centre, Salford Royal NHS Foundation Trust, University of Manchester, Manchester, UK

**Keywords:** Machine learning, Psoriatic arthritis

## Abstract

In view of the growth of clinical risk prediction models using genetic data, there is an increasing need for studies that use appropriate methods to select the optimum number of features from a large number of genetic variants with a high degree of redundancy between features due to linkage disequilibrium (LD). Filter feature selection methods based on information theoretic criteria, are well suited to this challenge and will identify a subset of the original variables that should result in more accurate prediction. However, data collected from cohort studies are often high-dimensional genetic data with potential confounders presenting challenges to feature selection and risk prediction machine learning models. Patients with psoriasis are at high risk of developing a chronic arthritis known as psoriatic arthritis (PsA). The prevalence of PsA in this patient group can be up to 30% and the identification of high risk patients represents an important clinical research which would allow early intervention and a reduction of disability. This also provides us with an ideal scenario for the development of clinical risk prediction models and an opportunity to explore the application of information theoretic criteria methods. In this study, we developed the feature selection and psoriatic arthritis (PsA) risk prediction models that were applied to a cross-sectional genetic dataset of 1462 PsA cases and 1132 cutaneous-only psoriasis (PsC) cases using 2-digit HLA alleles imputed using the SNP2HLA algorithm. We also developed stratification method to mitigate the impact of potential confounder features and illustrate that confounding features impact the feature selection. The mitigated dataset was used in training of seven supervised algorithms. 80% of data was randomly used for training of seven supervised machine learning methods using stratified nested cross validation and 20% was selected randomly as a holdout set for internal validation. The risk prediction models were then further validated in UK Biobank dataset containing data on 1187 participants and a set of features overlapping with the training dataset.Performance of these methods has been evaluated using the area under the curve (AUC), accuracy, precision, recall, F1 score and decision curve analysis(net benefit). The best model is selected based on three criteria: the ‘lowest number of feature subset’ with the ‘maximal average AUC over the nested cross validation’ and good generalisability to the UK Biobank dataset. In the original dataset, with over 100 different bootstraps and seven feature selection (FS) methods, HLA_C_*06 was selected as the most informative genetic variant. When the dataset is mitigated the single most important genetic features based on rank was identified as HLA_B_*27 by the seven different feature selection methods, consistent with previous analyses of this data using regression based methods. However, the predictive accuracy of these single features in post mitigation was found to be moderate (AUC= 0.54 (internal cross validation), AUC=0.53 (internal hold out set), AUC=0.55(external data set)). Sequentially adding additional HLA features based on rank improved the performance of the Random Forest classification model where 20 2-digit features selected by Interaction Capping (ICAP) demonstrated (AUC= 0.61 (internal cross validation), AUC=0.57 (internal hold out set), AUC=0.58 (external dataset)). The stratification method for mitigation of confounding features and filter information theoretic feature selection can be applied to a high dimensional dataset with the potential confounders.

## Introduction

Precision medicine has the potential to have an enormous impact on healthcare; however, for this potential to be fully realised, we need to be able to accurately predict the outcome of patients in different clinical scenarios. The wealth of genetic and clinical data that is now available for medical research provides an unprecedented opportunity to explore machine learning (ML) approaches for the prediction of the clinical outcomes^1,2^.

The use of genetic data in the development of risk prediction models presents a number of challenges mainly attributable to the large number of genetic variants available following imputation strategies and the high degree of redundancy between features due to linkage disequilibrium (LD). Many of these genetic variants may be completely irrelevant to the specific question being asked, or redundant in the context of other features. This may contribute to the increased computational burden of processing many similar features and the potential of overfitting to irrelevant aspects of the data. Therefore, it is important to identify a subset of the original variables (features) that enable more accurate prediction by the elimination of irrelevant or redundant information.

Filter methods, based on the information theoretic criteria, are particularly suited to these challenges as they are computationally less intensive than other methods, less likely to overfit and they evaluate the relationships of the features independent of any specific classifier^3^. In addition, information theory based on mutual information has the advantage of accounting for both linear and non-linear dependencies that exist between features whereas some traditional statistical methods such as logistic and lasso regression assume an additive genetic model^4,5^. This is of particular importance for many autoimmune diseases where genetic variants in the human leukocyte antigen (HLA) genes confer a substantial proportion of disease risk and studies have demonstrated highly significant non-additive effects^5^. In addition, the construction of a genetic prediction model may be confounded by issues such as population stratification, often represented by principal components, and ascertainment bias attributed to the method of sample collection. Here we explore the use of information theory based filter feature selection methods on HLA data to classify psoriatic arthritis (PsA)^6,7^ from cutaneous-only psoriasis. This is a clinically important question as approximately 30 percent of patients with psoriasis may develop PsA potentially leading to long-term disability and lower quality of life^8–10^. The ability to predict which psoriasis patients have a higher risk of developing PsA could lead to intervention strategies that would limit disability. We have previously shown that ascertainment bias in this data caused by the preferential collection of psoriasis cases with a young age of disease onset leads to confounding^11^ and here we illustrate the use of a stratification method to deal with such issues. Finally, we present an independently validated genetic prediction model based in information theoretic methods.

The following contributions are made:

- The development of a stratification approach to mitigate confounding
- We show that confounding features impacts the feature selection and can be successfully mitigated by stratification
- We demonstrate the utility of filter information theoretic methods for feature selection in highly complex genetic datasets such as the HLA region
- We present an externally validated risk prediction model for PsA using HLA data
  To our knowledge, no comparable techniques with stratification, information theoretic feature selection and external validation have been applied previously to the analysis of the HLA region in PsA and psoriasis

## Methods

### Sample cohorts

Our training dataset consisted of 1462 PsA patients recruited from rheumatology centres within in the UK. Classification of PsA was performed by a rheumatologist based on coexistence of psoriasis and inflammatory arthritis in accordance with the CASPAR (ClASsification criteria for Psoriatic ARthritis) classification system^12^ where possible. Recruitment was performed with full written informed consent from the patients (UK PsA National Repository MREC 99/8/84), all methods followed relevant guidelines and legistlation. This study was approved by Central Manchester NHS Research Ethics Committee. Data on 1132 cutaneous-only psoriasis patients (PsC) were available through the Biomarkers of Systemic Treatment Outcomes in Psoriasis study (BSTOP)^11^. Patients were recruited to BSTOP via the British Association of Dermatologists Biologics Interventions Registry (a UK pharmacovigilance registry, BADBIR.org.uk) from dermatology clinical within the UK. PsC classification in BSTOP is based on interview questioning of rheumatologist diagnosed PsA at baseline and follow-up visits (twice annually for the first three years of follow-up, then annually).

### Imputation of HLA alleles

**Figure 1:**
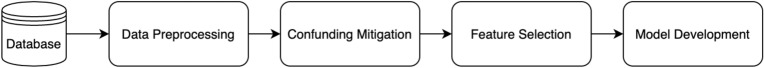
Research Pipeline.

Genotyping of DNA from PsA patients was performed with the Illumina Immunochip array as previously described^13^. Genotyping of DNA from PsC patients was performed at King’s College London using the Illumina HumanOmniExpressEx- ome-8v1-2_A as previously described^14^. Quality control was consistent across both dataset following conventional standards of data missingness (SNP and sample), SNP allele frequency, Hardy-Weinberg equilibrium and sample outliers based on relatedness and ancestry. Datasets were combined retaining an intersection of high quality SNPs. Quality control of UK Biobank genotype data is described in details by Bycroft et al^15^. Imputation of SNPs, amino acids and HLA alleles for training and validation datasets was carried out using SNP2HLA software package (version 1.0.3) using the T1DGC reference panel^11^. Variants with an information score < 0.9 or a MAF < 0.01 were excluded and all analyses were conducted using imputed dosage. Following quality control, the training dataset consisted of 2093 patients with 172 HLA alleles(2-digits,4-digits), 683 amino acids, 5862 SNPs. To ensure that our trained model is applicable to the validation data in UK Biobank, the data was filtered to only contain features that are shared between internal training cohort and external validation cohort. This analysis focuses on the 70 HLA 2-digits features and three potential confounders (age of psoriasis onset (aao), the top two principal components ’PC1’ and ’PC2’ for mitigation of population stratification^16^). The four stages research pipeline is illustrated in Fig. 1: data pre-processing, confounding mitigation, feature selection and model development for the subtype prediction.

### Stratification development

**Figure 2:**
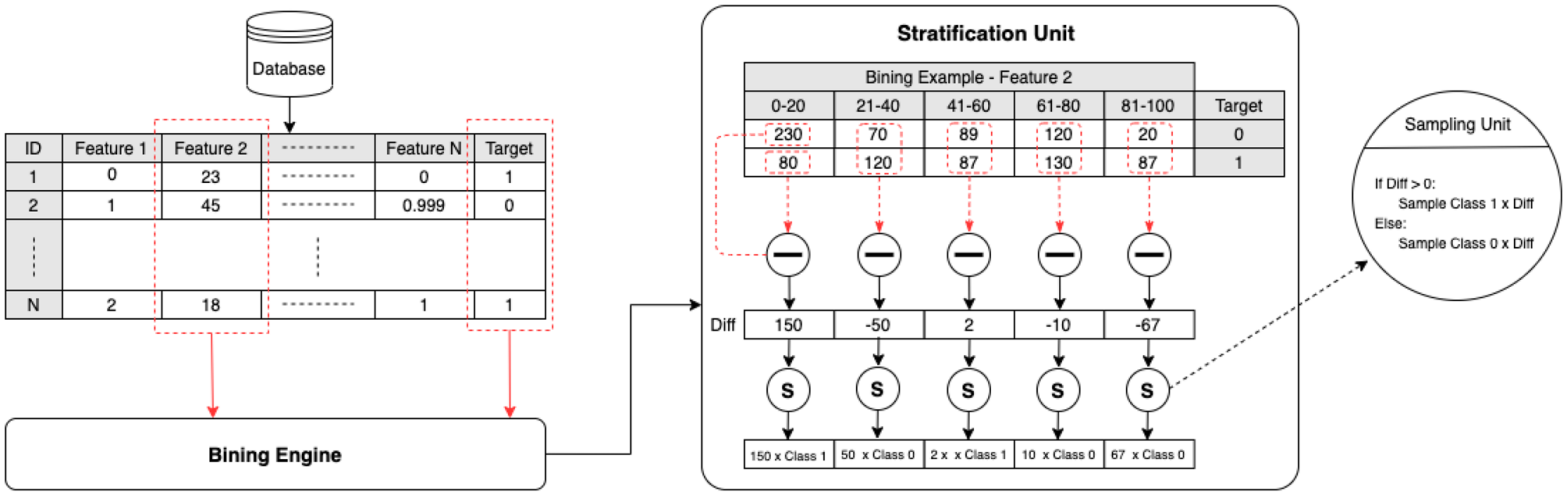
The potential confounders were mitigated by stratification.

To mitigate the effect of confounders in feature selection a ‘stratification’ method^17^ was developed to control for the three confounders of concern where the association between each feature and the outcome is tested within different strata of the confounding feature. During stratification, individuals are divided into several strata on the basis of confounders where the number of individuals in the strata may or may not be equal. Figure 2 illustrates the methodology of this approach for the stratification where ‘Feature 2’ has been assigned as the known age of psoriasis onset confounder. The minimum and maximum value for ‘Feature 2’ was determined and restricted to narrow width bins ‘0-20’ years ‘21-40’, ‘41-60’, ‘61-80’ and ‘81-100’. The Frequency distribution of each target label (‘PsC’=‘0’, ‘PsA’=‘1’) was balanced in each age boundary by random sampling with replacement(bootstrap)^18^. For instance, in Fig. 2 in ‘Stratification Unit’ the number of patients in age boundary ‘61-80’ for PsC is ten less than PsA patients and the frequency distribution of two classes with target 0 and 1 was balanced by the inclusion of 10 random samples in target class 0. The same procedure was applied to ‘PC1’ and ‘PC2’ where patients(n) are divided into two strata (− 1,0), [0,1).

### Information theoretic feature selection

Filter methods select features based on a performance measure regardless of the employed data modeling algorithm and separate the classification and feature selection components^3^. Filter methods are generally applied as pre-processing steps, with subset selection procedures that are independent of the learning algorithm and the defining component of filter based methods is scoring criterion, which is often as ‘relevance index’. The relevance index denotes how useful each feature is likely to be for the ML classification methods. Although this leads to a faster learning process, it is possible for the criterion used in the pre-processing step to result in a subset that may not work very well downstream in the learning algorithm. Univariate and multivariate methods are two categories for all filter based methods. Univariate methods, the scoring criterion only consider the relevancy of features while ignoring the feature redundancy. Mutual information is univariate feature selection approach (Shannon, 1948)^19,20^ measures the amount of information shared by an input feature X and class label (target) Y. Where the lower case x or y is possible values that the variables X and Y can adopt from the alphabet X and Y respectively in (1). To obtain this, we need to estimate the distribution of $p_{{\mathrm{x}}}$ and $p_{{\mathrm{y}}}$ respectively.

1 $$\begin{aligned} I(X;Y)= \sum _{x\in x}\sum _{y \in y} p(xy)log\frac{ p(xy)}{p(x)p(y)} \end{aligned}$$

Mutual Information Maximization (MIM) method given by (2) examines the mutual information between a class label Y and a feature $\hbox {X}_{{\mathrm{k}}},$ where *K* is the top features^21^. MIM assumes that all the features are independent and it does not account any dependencies between the features.

2 $$\begin{aligned} J_{\mathrm{{mim}}} (X_{{\mathrm{k}}})= I(X_{{\mathrm{k}}};Y) \end{aligned}$$

Multivariate method investigates the multivariate interaction within features and the scoring criterion is a weighted sum of feature relevancy and redundancy. The information theoretic methods investigate the multivariate interaction within features and the scoring criterion is weighted sum of feature relevancy and redundancy. Multivariate feature selection methods are described as follows. Joint Mutual Information (JMI) was proposed by Yang and Moody (1999)^22,23^. JMI is the information between the targets and a joint random variable defined by pairing the candidate $\hbox {X}_{{\mathrm{n}}}$ with each current feature. The redundancy term full captures by JMI.

3 $$\begin{aligned} J_{{\mathrm{jmi}}}(X_{{\mathrm{k}}})= \sum _{X_{{\mathrm{j}}}\in S}I(X_{{\mathrm{k}}}X_{{\mathrm{j}}};Y) \end{aligned}$$

Minimal-Redundancy-Maximal-Relevance (mRMR) given by (4) was proposed by Peng et al^24^. This takes the mean of redundancy term and it elemenated the conditional term. In equation (4), n is size of a feature set.

4 $$\begin{aligned} J_{{\mathrm{mrmr}}}(X_{{\mathrm{k}}})= I(X_{{\mathrm{k}}};Y) - \frac{1}{|S|}\sum _{{j}\in S} I(X_{{\mathrm{k}}};X_{{\mathrm{j}}}) \end{aligned}$$

Conditional Mutual Information Maximization (CMIM) given by (6) was proposed by Fleuret (2004)^25^ and is probably the most-well known recent criterion. CMIM measures the information between a feature and the target and it is conditioned on each current feature. The interaction information is the term in square brackets which can be both negative and positive. A negative value indicate that the shared information between $\hbox {X}_{{\mathrm{k}}}$ and Y has decreased as the result of including $\hbox {X}_{{\mathrm{n}}}$.

5 $$\begin{aligned} J_{{\mathrm{cmim}}}(X_{{\mathrm{k}}})=I(X_{{\mathrm{k}}};Y)- \underset{{X_{{\mathrm{j}}}}\in S}{max}[I(X_{{\mathrm{k}}};X_{{\mathrm{j}}})-I(X_{{\mathrm{k}}}; X_{{\mathrm{j}}}|Y)] \end{aligned}$$

Mutual information feature selection (MIFS) does not consider conditional redundancy (g = 0), but it does incorporate the redundancy penalty (Brown et al., 2012).

6 $$\begin{aligned} J_{{\mathrm{mifs}}}(X_{{\mathrm{k}}})=I(X_{{\mathrm{k}}};Y)-\beta \sum _{X_{{\mathrm{j}}}\in S}I(X_{{\mathrm{k}}}; X_{{\mathrm{j}}}) \end{aligned}$$

Double input symmetrical relevance (DISR) aims to better include such complimentary features by expanding JMI^26^. Disr normalises the information provided by a feature by how well the given feature complements the other features.

7 $$\begin{aligned} J_{{\mathrm{disr}}}(X_{{\mathrm{k}}})=\sum _{X_{{\mathrm{j}}}\in S}\frac{I(X_{{\mathrm{k}}}X_{{\mathrm{j}}};Y)}{H(X_{{\mathrm{k}}}X_{{\mathrm{j}}}Y)} \end{aligned}$$

The interaction capping (ICAP) approximated by following equation.

8 $$\begin{aligned} J_{{\mathrm{icap}}}(X_{{\mathrm{k}}})={I(X_{{\mathrm{k}}};Y)}-\sum _{X_{{\mathrm{j}}}\in S} max[0,\{I(X_{{\mathrm{k}}};X_{{\mathrm{j}}})-I[(X_{{\mathrm{k}}}; X_{{\mathrm{j}}}|Y)\}] \end{aligned}$$

We focus on seven filter feature selection (FS) methods: mutual information feature selection (MIFS), mutual information maximisation (MIM), joint mutual information (JMI), minimal-Redundancy-Maximal-Relevance (mRMR), conditional mutual information maximisation (CMIM), Interaction Capping (ICAP) and Double Input Symmetrical Relevance (DISR)^27^. We selected these methods based on computational efficiency, popularity in the literature and publicly available implementations, which increases their usability. The description of each feature selection can be found in supplementary section 1. Each feature is assigned a rank in order of their FS score and the top *K* features were selected. Predefined requirements for a certain number of features or other stopping criterion can inform the value of *K*^21,28^.

**Figure 3:**
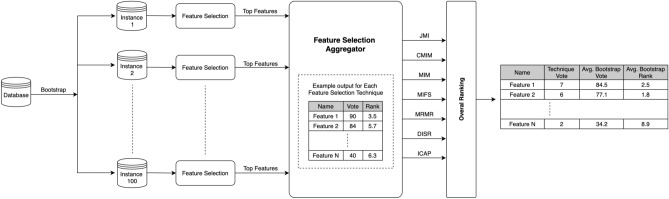
Methodology for feature selection.

Figure 3 shows our methodology for feature selection. We created 100 random samples with replacement (Bootstraps)^18^ from the original data and the top subset of features were obtained for each FS methods. For information theoretic criteria we estimated the necessary distributions using histogram estimators and features were discretised independently^21^. The HLA alleles were discretised to (0,1), [1,2) and PC1, PC2 to (− 1,0), [0,1). In ‘Feature Selection Aggregator’ the outputs vote ‘V’ and rank ‘R’ are generated respectively. The vote ‘V’ for a features criteria defines the majority voting over 100 bootstraps. The rank ‘R’ defines the average rank over 100 bootstraps as the rank of each feature in the top selected features can vary in each bootstrap.

Once this ranking has been computed, a feature subset composed of the best feature subset was created. For instance, Feature 1 with vote=90 and rank ‘R=3.5’ over 100 bootstraps is selected as the top ‘1’ feature subset. We incrementally selected top features ranging from (n=1,10, … 70) using each of the seven feature selection methods. This subset of selected features was then used as an input to each of seven supervised machine learning (ML) algorithms.

The top selected feature subset may vary with respect to each FS criterion. We therefore proposed overall ranking Fig. 3 with the ‘Technique Vote’, the ‘Average Bootstraps Vote’ (ABV) and ‘Average Bootstrap Rank’(ABR) that explore the rank of features across ‘seven different FS techniques’. The ‘Technique Vote’ is a selection of feature by FS criteria. The ‘Average bootstraps vote’ and ‘Average bootstrap rank’ are defined by equations 9 and 10 respectively.

9 $$\begin{aligned} \text{ ABV }= \frac{\text{ The } \text{ sum } \text{ of } \text{ votes } \text{ number } \text{(v) } \text{ in } \text{ each } \text{ FS } \text{ criteria }}{\text{ Number } \text{ of } \text{ FS } \text{ techniques }} \end{aligned}$$

10 $$\begin{aligned} \text{ ABR }= \frac{\text{ The } \text{ sum } \text{ rank } \text{ of } \text{ feature } \text{(R) } \text{ in } \text{ each } \text{ FS } \text{ criteria }}{\text{ Number } \text{ of } \text{ FS } \text{ techniques }} \end{aligned}$$

All these feature selection methods are compared with the case when all of the 70 HLA 2-digits features are fed into the classifier for prediction. All feature selection methods are publicly available from the package skfeature(1.00) feature open access repository of Python(3.6.10) programming language which provides individual rankings to each feature in the database.

### Supervised risk prediction model development and internal validation

In this study, risk prediction models for PsA were developed using seven supervised ML algorithms:^29–31^ Logistic Regression (LR), AdaBoost, XGBOOST, Random Forest (RF), K-nearest neighbor classifier (KNNC), Decision Tree (DT) and Gaussian naive bayes (NB)^29^. For each feature subset the response measurement is PsA (class=1) and PsC (class=0). The original set of examples provides the training data and the learning algorithm has been trained and validated using stratified nested cross-validation. Many of the machine learning algorithms employed have one or more hyper-parameters that must be selected to optimise model performance. The most optimal hyperparameter for each ML model have been obtained using 5-2 fold nested cross validation stage.The purpose of our nested cross-validation was to find an unbiased view of the overall expected performance of each model, so the hyperparameter tuning process in this step only help to find the most accurate version of each algorithm in each nested-cross validation fold. The aim in this stage is only to evaluate and compare the learning power of each algorithm using different folds of data and removing any bias in the process. Thus, to achieve the optimal hyperparameters for each ML model we re-trained and performed a hyperparameter tuning for all the algorithms using the entire training data set. These hyperparameters are then used to evaluate the performance of each model on the test dataset.

### External validation of risk prediction models

**Figure 4:**
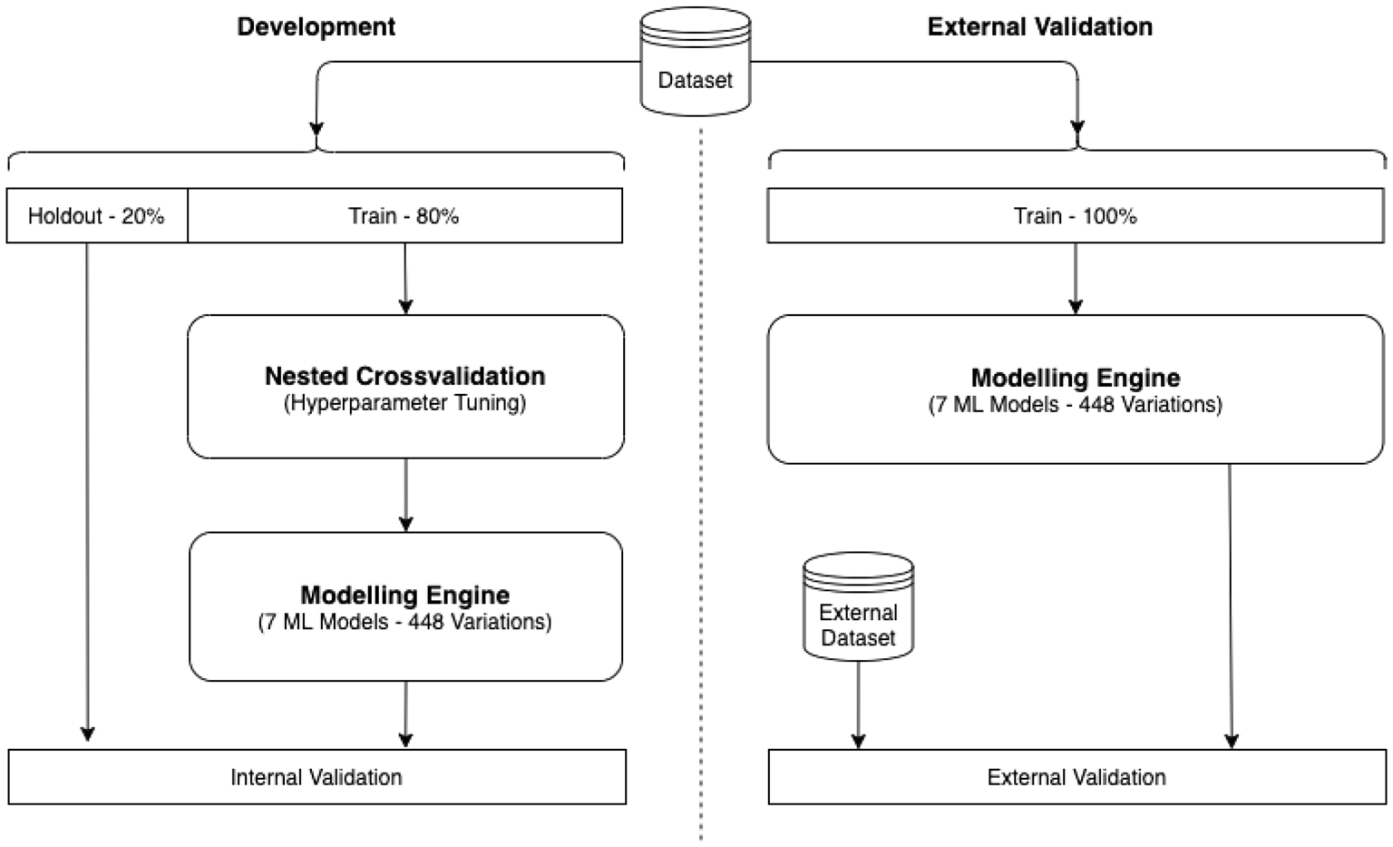
Risk prediction model development and external validation.

Ultimately, fully independent external validation with data available at the time of PsA prediction development is important. Here we use data from UK Biobank for external validation to test the generalisability of seven developed ML classifiers on PsA data. The assessment is for reproducibility rather than transportability as the external data is very similar to the PsA-MD data set^32^. Figure 4 presents our pipeline for internal model development and external validation. 80% of data was randomly used for training of ML classifiers using 5-2-fold stratified nested cross validation and 20% was selected randomly as a holdout set for internal validation.

There are 448 different models with post mitigation trained using a combination of ‘number of features (1,10,...70)’, ‘7 feature selection methods’, ‘7 ML Model type’. We used the whole data and the optimal hyperparameter to test the best generated models in the UK Biobank dataset. Therefore, for each ML models 448(all models)/7(MLmodels)= 64 different combinations have been generated and the models with the maximal average AUC in nested cross validation is selected as the best model and tested for the external validation.

All machine learning analyses were performed in Python (using the numpy, pandas, sklearn, matplotlib, and XGBOOST packages), which provides a user-friendly interface to access many machine-learning algorithms in Python. We used AUC, precision recall curve, precision (positive predictive value (PPV)), recall (true positive rate or sensitivity) and F1 score to evaluate the performance of the ML classifiers.

## Results

We developed a stratification method to control for known confounding for population stratification (two principal components) and age of psoriasis onset. We then used a range of information theory feature selection methods and ML supervised classification methods to develop a risk prediction model for classifying PsA from cutaneous-only psoriasis. The best model was then externally validated in an independent dataset from UK Biobank to assess the generalisability of the predictive performance.

### Impact of confounding on feature selection

**Figure 5:**
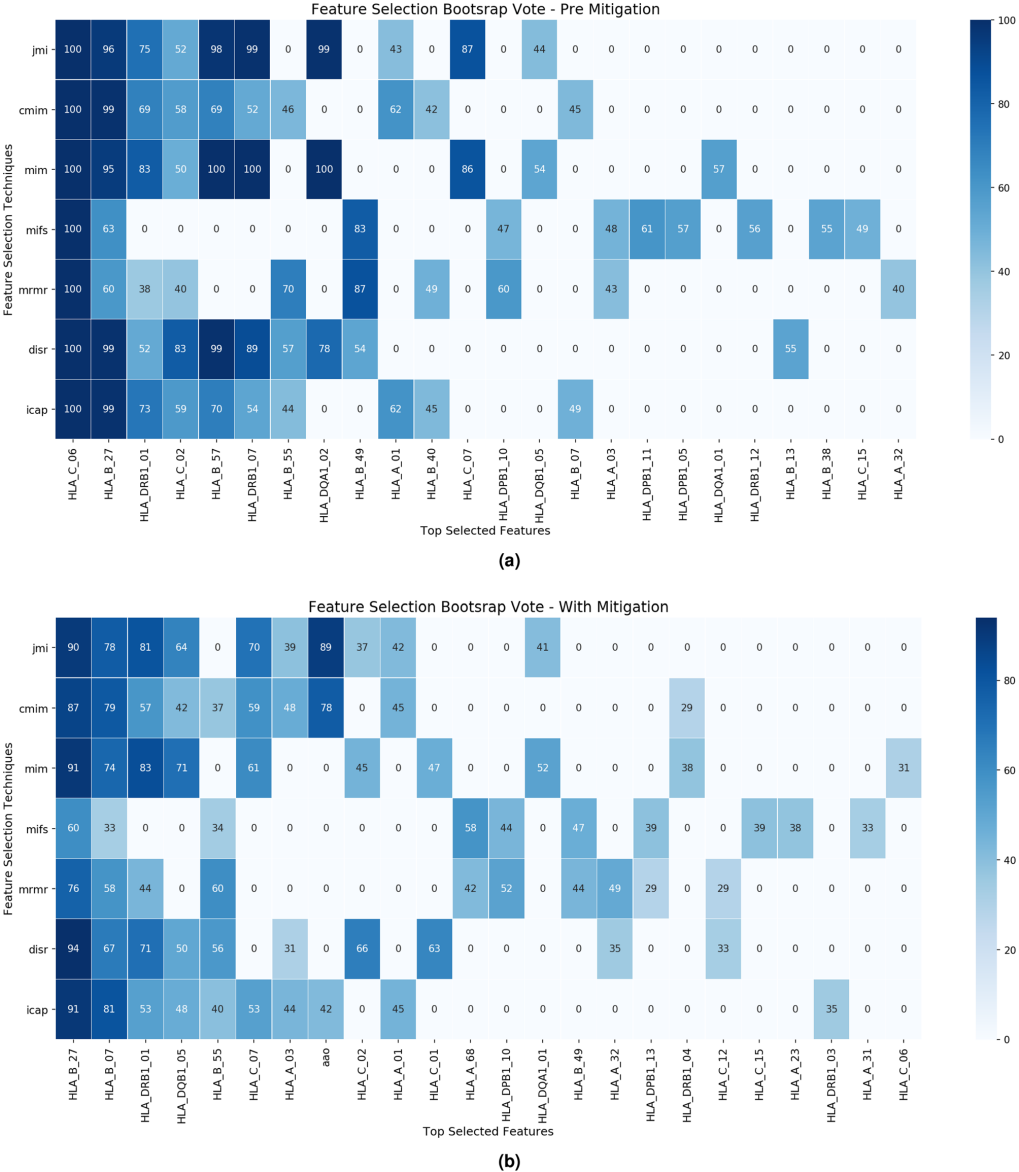
Heatmap (**a**) feature ranking in original dataset, Heatmap (**b**) of feature ranking post-mitigation depicting the majority vote over 100 bootstrap. The top 10 selected features (in rows) and seven features selection techniques in (columns).

We investigated the impact of confounding on feature selection pre and post-mitigation by stratification using seven FS information theoretic criteria methods MIFS, MIM, JMI, mRMR, CMIM, ICAP and DISR. The FS information theoretic methods were applied to a dataset of 1462 PsA cases and 1132 cutaneous-only psoriasis cases^13^ using 2-digit 70 HLA alleles. Figure 5a,b illustrate the top 10 selected features for the seven FS criteria and its vote over 100 bootstraps^18^ pre and post-mitigation for the three potential confounders.

HLA_C_*06 is the genetic variant that makes the largest contribution to psoriasis susceptibility and is known to be highly correlated with age of psoriasis onset^11^. Over 100 different bootstraps and seven FS methods HLA_C_*06 is selected as the most informative genetic variant in the original dataset Fig. 5a. After the mitigation of the three potential confounders, HLA_B_*27 had vote 94 for ‘DISR’ followed by HLA_B_*07 with the vote ‘81’ in ‘ICAP’ and HLA_DRB _01 with the vote ‘83’ in Fig. 5b. PC1 and PC2 were not observed in any of the top 10 features subset and the majority vote over 100 bootstraps for ‘age onset’ had dramatically dropped after the stratification mitigation Fig. 5b. The results demonstrate how confounding impacts the selected features and that the stratification mitigates this impact and this is clearly illustrated by the absence of HLA_C_*06 following the mitigation.

### Impact of confounding on classification and internal validation

For ML classification we have used dynamic and fixed number of features where the main aim was to identify a subset of features which maximize the risk prediction model performance in the internal dataset and generalisability to UK Biobank as the external validation set. In order to compare the performance of different models on a varying number of features with feature step (1, 10,...70), the top feature subset were consecutively incorporated into each model pre mitigation with no confounders and post mitigation of three confounding features.

**Figure 6:**
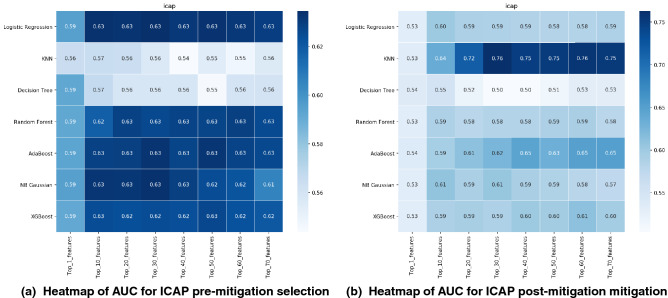
The ICAP feature selection pre-mitigation and post-mitigation for seven classification methods. Heatmap depicting the predictive performance (AUC for hold out set) for different number of HLA features(in rows) and different classification method in (columns).

Figure 6 shows the AUC for hold out set for ICAP feature selection for all the seven models ‘pre mitigation with no confounders’ and ‘post mitigation with three mitigated confounders’. It can be observed for ICAP feature selection all classifiers show similar predictive performances. There is $\approx$10% drop in the performance of classification post mitigation of confounding features. In pre-mitigation, the highest performance around 0.63 was obtained for LR, RF AdaBoost and NB Gaussian when the top 20 subset of features selected by ICAP were incorporated into these models. In the post mitigation, the AUC was 53% for the top 1 HLA feature, which was improved by $\approx$20% for 20 features in KNNC and there is drop in the AUC for other classifiers in the post mitigation. In conclusion, adding more features to the models did not improve the AUC dramatically in pre and post mitigation (except KNNC that showed different behaviour). The Figures 2, 3, 4, 5 in supplementary were generated for all other feature selection methods in pre and post mitigation with obtained AUC in nested cross validation and hold out set. All the feature selection and classifiers show similar behaviours as ’ICAP’. The results of overall ranking of feature selection can be found in Figures 2, 3, 4 and 5 in supplementary

### External validation of classification models

The risk prediction models were then further validated in the UK Biobank data set containing data on 1187 participants and a set of features overlapping with the training dataset. The validation data set had similar characteristics, although a lower proportion of admissions from the patients with PsA and PsC. Table 1 presents the comparison results of AUC, precision and recall of the best generated models out of 448 different models. The best models number are 402(LR), 303 (Adaboost), DT (416), XGBoost(398), KNNC (232), NB Gussain(39)and RF(184).The performance is dependent on the type of feature selection methods, the number of selected features and the selected prediction models. The results for accuracy and F1 score of the best models can be found in Table 1 supplementary.

**Table 1:** The best generated models out of 448 generated models.

	The best models			AUC %			Precision%			Recall%		
Model Num	Model name	Feature Selection	Top features	Cross validation	Hold out	External	Cross validation	Hold out	External	Cross validation	Hold out	External
402	LG	disr	40	0.62	0.58	0.57	0.60	0.53	0.55	0.57	0.54	0.57
303	Adaboost	jmi	60	0.66	0.64	0.54	0.62	0.59	0.52	0.61	0.64	0.60
416	DT	disr	10	0.54	0.53	0.51	0.67	0.63	0.53	0.16	0.16	0.20
398	XGBoost	disr	40	0.63	0.60	0.55	0.60	0.56	0.53	0.57	0.53	0.56
232	KNNC	disr	60	0.73	0.76	0.53	0.73	0.74	0.53	0.74	0.76	0.52
39	NB Gussain	mim	10	0.61	0.58	0.59	0.63	0.54	0.57	0.35	0.34	0.42
184	RF	icap	20	0.61	0.58	0.58	0.59	0.54	0.58	0.54	0.45	0.59

**Figure 7:**
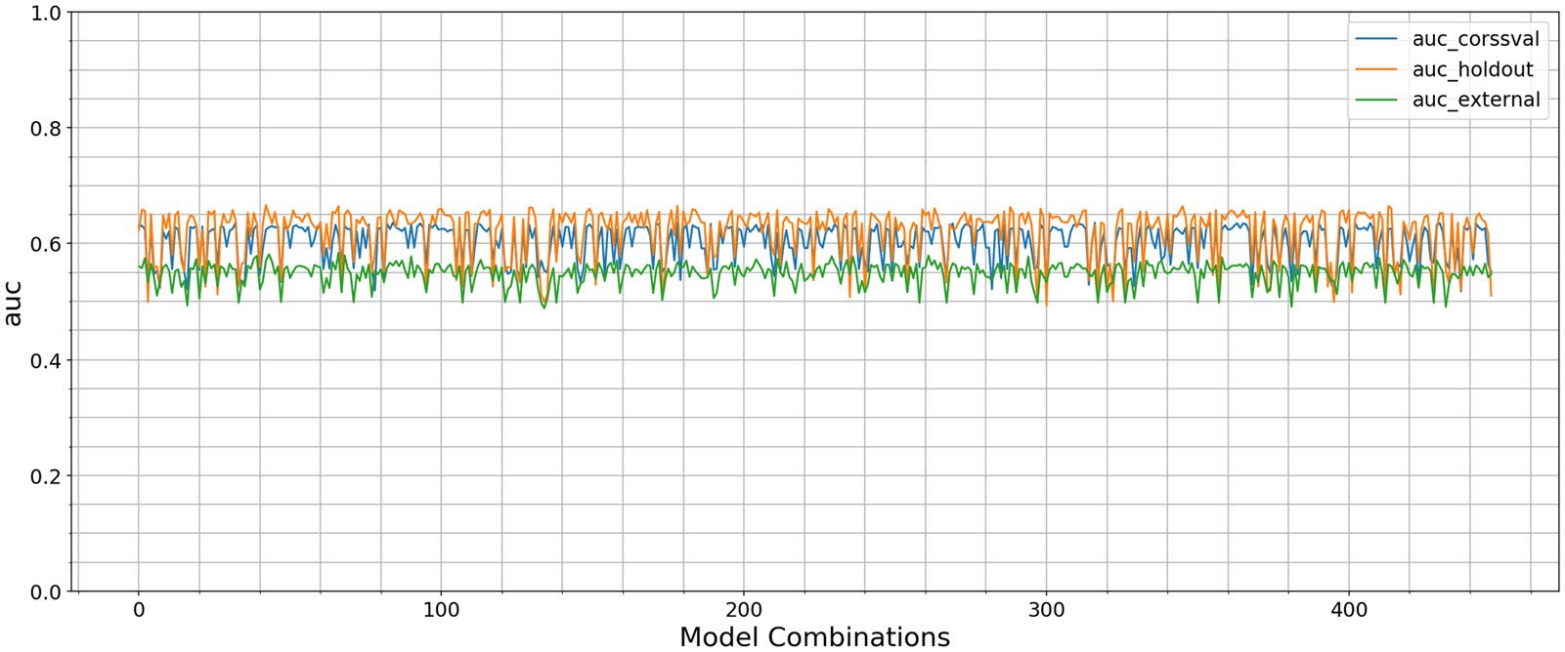
Comparison between AUC for cross validation, hold out and external set for 448 different generated ML models.There are 448 models trained using combination of ‘number of features’, ‘Feature Selection’, ‘7 ML Model type’.

All models for predicting risk of PsA demonstrated moderate predictive performance. In post mitigation, a RF model with 20 features selected by ICAP performed as the final best overall model. RF has good generalisibility and robustness with respect to internal-cross validation (AUC= 0.61, Precision=0.59, Recall=0.54), internal-hold out-set(AUC= 0.58, Precision=0.54, Recall=0.45) and external validation (AUC= 0.58, Precision=0.58, Recall=0.59). Amongst all models KNNC (model number=232, disr, 60 features) is overfitted in UK biobank with AUC internal (cross validation= 0.73, hold out= 0.76) and (AUC external=0.53). Of note, Gussain (model number=39, mim, 10 features), DT (model number=416, disr, 10 features) have very similar AUC to RF but lower precision and recall. Each feature selection and classification model combination have different behaviours to the mitigation techniques. Variability in the classification models and feature selection methods were the main factors in the performance variation. Overall, classification AUC at the model development stage were comparable to AUC where those models were used to predict labels in UK biobank as shown in Fig. 7. Nested cross validation was sufficient to control overfitting and produced results which generalised well to the independent test sample. The hyperparameters for each classifier is shown in Table 2.

**Table 2:** Machine Learning Algorithms and their Corresponding Hyperparameters.

Model	Scikit-Learn package	Parameter name in Scikit_Learn Package	Test Range
DT	tree.DecisionTreeCalssifier	max_features	[1, 10, 20, 30, 40, 50, 60, 70]
		max_depth	[1, 2]
		min_sample_split	[2, 5, 10]
		min_sample_leaf	[2, 3, 4, 5]
XGBoost	xgboost.XGBClassifier	n_estimators	[100, 200, 300, 400]
		learning_rate	[0.1, 0.5, 1.0]
		max_depth	[1, 2]
		min_child_weight	[1, 3]
		eta	[0.8]
		gamma	[2]
		lambda	[0.5]
		alpha	[0.5]
RF	ensemble.RandomForestClassifier	n_estimators	[100, 200, 300, 400]
		max_depth	[1,2]
		max_feature	[1,10,20,30,40,50,60,70]
		min_sample_leaf	[2,3,4, 5]
		min_samples_split	[2,5,10]
AdaBoost	ensemble.AdaBoostClassifier	n_estimator	[100, 200, 300, 400]
		learning_rate	[0.1, 0.5, 1.0]
LR	linear_model.LogisticRegression	C	[0.01,0.1,1,10]
KNN	neighbors.KNeighborsClassifire	K	[1, 3, 5]
NB Gaussian	naive_bayes.GaussianNB	—	—

**Figure 8:**
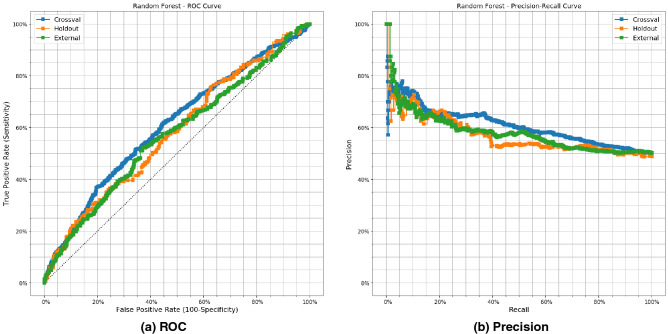
(**a**) Receiver operating characteristic and (**b**) Precsion-Recall curve of the best models with internal( hold out and cross validation) and external model performance for RF.

**Figure 9:**
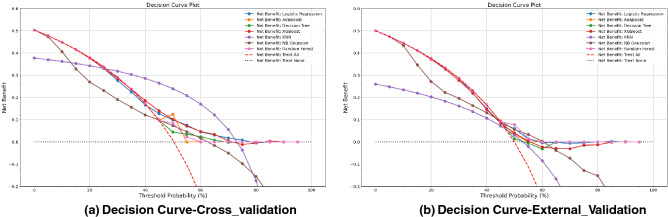
Decision curve analysis for seven machine learning models for prediction of psoriatic arthritis (PsA).

The Figures 8 and 9 in supplementary generated for accuracy, precision, recall and F1-score score of 448 generated models and each model respectively. Figure 8 shows Receiver operating characteristic (ROC) curve and precision-recall (PR) curve for predicting PsA. ROC curve of internal validation (cross validation, holdout-set), external validation (a) and PR curve of internal validation (cross validation, hold out-set) and external validation (b)illustrate that the best classifier RF. The results of ROC curve and PR are generated for other classifiers in supplementary Fig. 6 and Fig. 7 respectively. Figure 9a,b show the positive net benefit for each model within a specific threshold in cross validation and external validation. Probability threshold $\hbox {p}_{{\mathrm{t}}}$ in the studied population is between 25% and 75%. KNNC has the positive net benefit between [25%–75%] in cross validation but its performance drops, measured by AUC, as its shown in Table 1. We can observe that for our best model ‘Random Forest’ a positive net benefit between 45% and 60% threshold probability. The decision curve is generated for each model in cross validation and external datast in Fig. 10 supplementary.

## Discussion

Our results clearly demonstrate the impact of confounding on both feature selection and model generalisability and how stratification can mitigate this effect. This is well illustrated by considering the results for HLA_C_*06, which is a known risk factor for early onset psoriasis (type I): we have previously shown the preferential collection of type I psoriasis in genetic studies can lead to ascertainment bias when compared with PsA. Our stratification approach mitigated this effect leading to the expected identification of HLA_B_*B27 as the predominant risk factor for PsA in psoriasis.

The issue of selection bias and confounding is increasingly being recognised as an important issue in the statistical methodology literature^33,34^. The main aim of this work was to demonstrate the application of information theory methods to genetics, in particular for feature selection in complex regions of the genome, namely the MHC, and in the presence of confounding. The results from our external validation confirm the generalisability and reliability of the AUC values obtained in the training data following mitigation by stratification. In addition, high dimensional data with many redundant features, such as found with genetic datasets, are a significant challenge for Machine Learning^35^. Our results demonstrate the utility of information theory feature selection methods in complex genetic datasets, such as the HLA region, which, coupled with the fact that they are less prone to overfitting and computationally efficient, makes them attractive options for feature selection in genetic datasets.

Our study has several strengths: firstly, we aimed to avoid overfitting in two stages, initially at the internal stage where we used stratified nested cross validation and tested each model on unseen data (hold out set) and subsequently we externally validated the best models on a completely independent dataset. Secondly, our feature selection method is independent of classification methods and it does not assume an additive or linear relationship between the features and the outcome. Many prediction models and risk scores have been developed with feature selection methods based on traditional statistical approaches such as logistic and lasso regression. The traditional methods will fit better if the data is linearly separable^36,37^. If such a linear relationship does not exist then the model may oversimplify complex relationships among features with non-linear interactions, leading to the potential loss of significant relevant information^38,39^ which is likely to be the cases in the HLA region for many autoimmune diseases where non-additive effects have been reported^5,40^.

The moderate performance of our prediction model in internal and external validation could be explained by the fact that the imputed HLA alleles are not sufficient to differentiate PsA from cutaneous-only psoriasis despite this being the major PsA genetic risk factor^41^.

In addition, the sample size and cross-sectional nature of the dataset may also be a limitation for a training machine learning algorithm where the performance of risk prediction model based on ML classifiers will be better if the number of training samples is large^42^. The research looked at the genetic variants found in the MHC region, so, the genetic variants outside of the MHC region may improve the prediction models performance. Combination of clinical data and genetics data can be used in a longitudinal fashion to improve performance.

We used classic information theoretic methods, so using two state of art models in information theory may improve the performance of the models and the selection of informative features.

Finally, whilst doing our utmost to ensure that the cutaneous-only psoriasis reference groups were free from PsA there is the potential for phenotype misclassification where a proportion of these participants have gone on or will go on to develop PsA. The BSTOP patients are screened for PsA with the use of a questionnaire which is not as efficient as screening by a rheumatologist. In this study, we can assume a certain level of undiagnosed PsA in the PsC group which will impact the classification accuracy^43,44^. In general larger number with clearly characterise is important, both for PsC and PsA . In PsC, area of involvement, as well as overall Psoriasis Area and Severity Index (PASI) and nail disease should be taking into consideration.This would impact both model training and external validation.

An ML algorithm is considered non generalisable and unstable if a small change in the training set causes a large change in the performance of the algorithm^45^. The more stable an algorithm, the more reliable are its results and the greater the confidence in the results. It is not adequate for an ML algorithm to perform well on a hold out test dataset, ideally it must also be stable and generalisable to external dataset. To our knowledge this is the first study to explore the application of information theoretic feature selection methods to genetic data. A recent study exploring machine learning methods for the prediction of PsA reported an AUC of 0.58 in cross-validation and 0.54 on the training dataset using five HLA variants. We have used the established ‘classic information theoretic methods’ which have currently available libraries. Two state of art information theoretic methods ‘Feature selection considering Uncertainty Change Ratio of the Class Label’ and ‘Feature redundancy term variation for mutual information-based feature selection’ may improve the performance of the prediction models^46^. In conclusion, our study demonstrates the ability of stratification approach to mitigate the impact of confounding and we present an externally validated model based on data from the HLA genes for predicting risk of PsA in patients with psoriasis.

## Conclusion and future work

This study showed the ability of stratification, filter feature selection methods and machine learning to identify risk factors and predict outcome across genetic data, which should lead to greater insights on disease risk factors with no prior assumption of causality. To our knowledge this is the first study to assess the impact of confounders on feature selection using information theoretic methods and characterise the risk of developing PsA using of machine learning algorithms in a UK psoriasis population. Further validation of the developed methods with different clinical outcomes, different biomarkers, wider spectrum of genetic variables and also different PsA cohorts could provide better insights about their applicability. Future research in the area should move towards combining clinical data and genetics in a longitudinal manner for better prediction of the outcome. MIFS, MIM, JMI, mRMR, CMIM, ICAP, and DISR are seven classic approaches for feature selection employed in the proposed methodologies. The proposed stratification and machine learning methods should be compared to two state of art methods models: ‘Feature selection considering Uncertainty Change Ratio of the Class Label’^47^ and ‘Feature redundancy term variation for mutual information-based feature selection’^48^ as the future work.

## Supplementary information

Supplementary Information 1.

## Data Availability

Source codes of the programmes and algorithms used for this study are available from the corresponding author upon reasonable request.
