# Supplementary material for: Application of information theoretic feature selection and machine learning methods for the development of genetic risk prediction models
Source: Sci Rep. 2021 Dec 2;11:23335. doi: 10.1038/s41598-021-00854-x (PMC8640070; doi:10.1038/s41598-021-00854-x)
Supplement: Supplementary file 1 — Supplementary Information 1. [file 41598_2021_854_MOESM1_ESM.pdf]

Supplementary

Application of information theoretic feature selection and machine learning methods for the development of genetic risk prediction models

1 Dimensionality reduction

Dimensionality reduction is the process of reducing the number of features that need to take into account when making predictions. Dimensionality reduction methods can be classified as 'feature extraction' or 'feature selection' methods. Feature extraction methods transform a high-dimensional space of feature measurements to a space of fewer dimensions. Principal Component Analysis (PCA) can achieve such a transformation without reference to the output scores by exploiting dependencies between feature measurements, that may be assumed to be linear[1]. Many other transformations exist which can exploit nonlinear dependencies and the statistics of the output scores. 'Feature selection' approaches try to find a subset of the original variables that enable more accurate prediction by the elimination of irrelevant and confusing information. Filter methods select features based on a performance measure regardless of the employed data modeling algorithm and separate the classification and feature selection components. Filter methods are generally applied as pre-processing steps, with subset selection procedures that are independent of the learning algorithm and the defining component of filter based methods is scoring criterion, which is often as 'relevance index'[2]. The relevance index denotes how useful each feature is likely to be for the ML classification methods. Although this leads to a faster learning process, it is possible for the criterion used in the pre-processing step to result in a subset that may not work very well downstream in the learning algorithm. The information theoretic methods investigate the multivariate interaction within features and the scoring criterion is weighted sum of feature relevancy and redundancy. The main goal of feature selection is obtaining a subset of features that produces the highest 'Area under the ROC Curve' (AUROC) and the precision-recall curve on the classification models[3]. It has been mathematically proven that the performance ranks of two models remain same in the ROC space and the PR space[4]. The classification performance is necessarily proportional to removal of redundant features. Wrapper methods[1,5] search the space of feature subset based on the accuracy of a particular classifier (e.g. LR or RF). Embedded methods perform feature selection in the process of training and during the modeling algorithm's execution. Hybrid methods were proposed to combine the best properties of filters and wrappers. Direct feature selection searches to identify, individually, the relevant features and discard the irrelevant ones. Such methods are instances of a wide range of general strategies for dimensionality reduction, which seek to map the input variables into a lower dimensional space prior to running the supervised learning algorithm. A learning algorithm is faced with the problem of selecting a relevant subset of features which makes the best prediction while ignoring the rest in the features. Since the usual goal of supervised learning algorithms is to minimise regression error on an unseen test set, we have adopted this as our goal in guiding the feature subset selection. Univariate and multivariate methods are two categories for all filter based methods. Univariate methods, the scoring criterion only consider the relevancy of features while ignoring the feature redundancy. Multivariate method investigates the multivariate interaction within features and the scoring criterion is a weighted sum of feature relevancy and redundancy.

2 Nested Cross Validation

Nested cross validation was used at model development stage to assure good generalisability when the models were tested with hold out data. Varma and Simon[6] report a bias in error estimation when using cross-validation for model selection; therefore, we used stratified nested cross-validation as an almost unbiased estimate of the true AUC. The validation data-set is often used to fine-tune models. For example, we try out various sets of K for a KNNC model by finding the AUC produced by each set of K for the validation data-set. This would allow us to choose among the competing sets of K. In such a case, the AUC with the validation data-set will be an optimistic estimate of how the fine-tuned model would perform with unseen data[1]. This is because the final K will has been chosen such that the AUC with the validation data-set is the highest possible.

BADBIR Study Group

BADBIR Study Group

BADBIR Robert Chalmers[1], BADBIR Carsten Flohr [2], BADBIR Karen Watson[3], BADBIR David Prieto-Merino [4], BADBIR Gabrielle Becher[5], BADBIR Anthony Bewley [6], BADBIR David Burden[7], BADBIR Simon Morrison[8], BADBIR Phil Laws[9], BADBIR Ian Evans[10], BADBIR Christopher Griffiths[11], BADBIR Shehnaz Ahmed[12], BADBIR Brian Kirby[13], BADBIR Elise Kleyn[14], BADBIR Linda Lawson[15], BADBIR Teena Mackenzie[16], BADBIR Tess McPherson[17], BADBIR Kathleen McElhone[18], BADBIR Ruth Murphy[19], BADBIR Anthony Ormerod[20], BADBIR Caroline Owen[21], BADBIR Nick Reynolds[22], BADBIR Amir Rashid[23]
[1]The University of Manchester
[2]King's College London and Guy's and St. Thomas' NHS Foundation Trust
[3]Consultant Dermatologist in Sawbridgeworth, Hertfordshire
[4]London School of Hygiene and Tropical Medicine
[5]NHS Greater Glasgow and Clyde
[6]Consultant Dermatologist Whipps Cross Hospital and Royal London Hospital
[7]Department of Dermatology, University of Glasgow, Glasgow, United Kingdom
[8]Chief Executive Officer
[9] MBChB The Leeds Teaching Hospitals NHS Trust
[10]Dermatology Centre, Salford Royal NHS Foundation Trust; The University of Manchester, Manchester Academic Health Science Centre, Manchester, U.K.
The Dermatology Centre, Salford Royal NHS Foundation Trust, The University of Manchester, Manchester Academic Health Science Centre, Manchester, UK
[11]MD, FMedSci The University of Manchester
[12]Director of Research and Publishing, British Association of Dermatologists
[13]St Vincent's Private Hospital Merrion Road
[14]The university of Manchester
[15]BADBIR Team
[16]RN, BSc Oxford University Hospitals
[17]MD (University of Oxford)
[18]The University of Manchester
[19]PhD Nottingham University Hospitals
[20]University of Aberdeen
[21]East Lancashire Hospital NHS Trust
[22]Medical School Newcastle University
[23]The University of Manchester

BSTOP Study Group

BSTOP David Burden [1], BSTOP Stefan Siebert[2], BSTOP Sara Brown[3], BSTOP Helen McAteer[4], BSTOP Julia Schofield [5]
[1]Department of Dermatology, University of Glasgow, Glasgow, United Kingdom;
[2]Institute of Infection, Immunity and Inflammation, University of Glasgow, Glasgow, United Kingdom
[3]Skin Research Group, School of Medicine, University of Dundee
[4]Chief Executive of the Psoriasis Association
[5]United Lincolnshire Hospitals NHS Trust

3 Figure for original dataset with potential confounders

We added three potential confounders aao,PC1 and PC2 to the dataset and no mitigation is applied to the confounders in Figure 1. aao had the vote 100 for all features selection criteria and followed by HLA_B _*06 with the vote '100' in 'MIM' and JMI

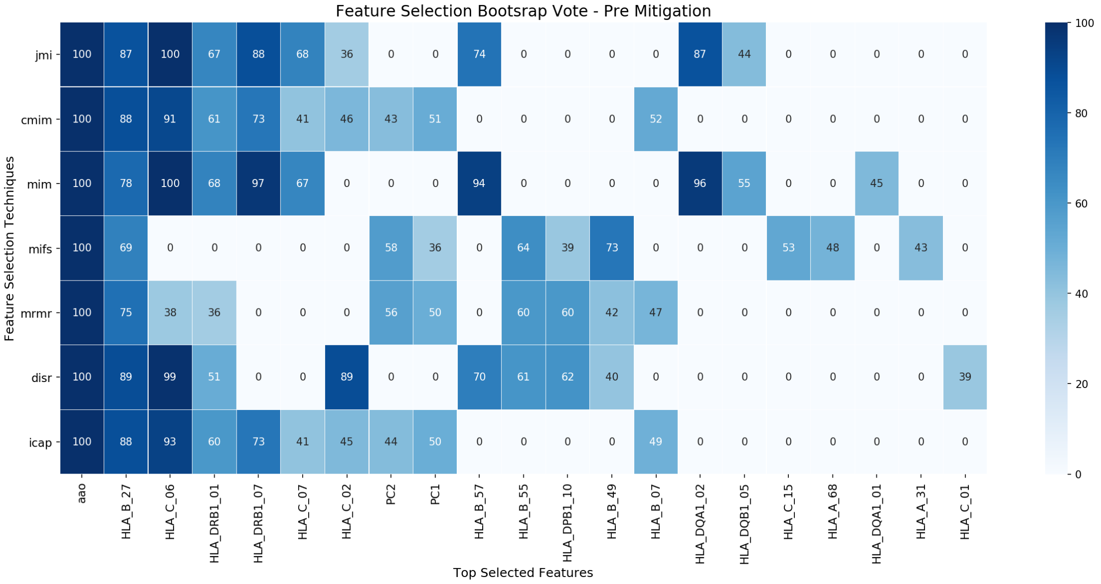

Figure 1. Heatmap (a) feature ranking unmitigated the majority vote over 100 bootstrap mitigation of aao, PC1, PC2 for the top 10 selected features (in rows) and seven features selection techniques in (columns)

and HLA_DRB1_07 with the vote '97' in 'mim'and HLA_B_*27 with the vote '89' in 'disr. PC1 and PC2 had the average vote for the most FS criteria and there effect was mitigated post to the mitigation.

4 Figures for impact of unmitigated confounding on feature selection

Figure 2 and Figure 3 show the average AUC in nested cross validation and AUC in hold out set respectively when CMIM, DISR, ICAP, JMI, MIFs, MRMR and overall ranking was applied to the original dataset.

5 Figures for impact of mitigated confounding on feature selection

Figure 4 and 5 illustrate the average AUC in nested cross validation and the AUC in hold out set respectively when CMIM, DISR, ICAP, JMI, MIFs, MRMR and overall ranking was applied to the mitigated dataset

6 Figures for evaluation metrics

Figures 6,7 show ROC curve and precision-recall for 6 ML models

Figures8 show the accuracy, precision, recall and F1 score for 448 generated different models. Figures 9 depict the 64 different combination for each ML model.

References

1. Jalalinajafabadi, F. *Computerised GRBAS Assessement of Voice Quality*. Ph.D. thesis, The University of Manchester (United Kingdom) (2016).
2. Guyon, I. & Elisseeff, A. An introduction to variable and feature selection. *J. machine learning research* **3**, 1157–1182 (2003).
3. Das, S. Filters, wrappers and a boosting-based hybrid for feature selection. In *Icml*, vol. 1, 74–81 (2001).
4. Davis, J. & Goadrich, M. The relationship between precision-recall and roc curves. In *Proceedings of the 23rd international conference on Machine learning*, 233–240 (2006).
5. Kohavi, R., John, G. H. *et al.* Wrappers for feature subset selection. *Artif. intelligence* **97**, 273–324 (1997).
6. Varma, S. & Simon, R. Bias in error estimation when using cross-validation for model selection. *BMC bioinformatics* **7**, 91 (2006).

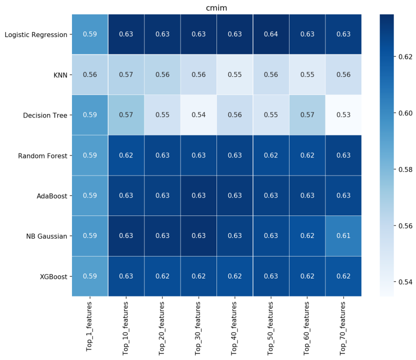

(a) CMIM

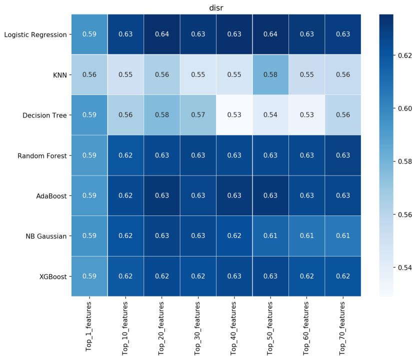

(b) DISR

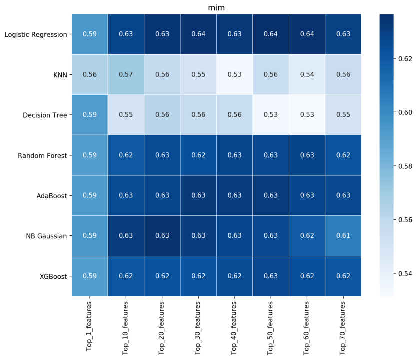

(c) mim

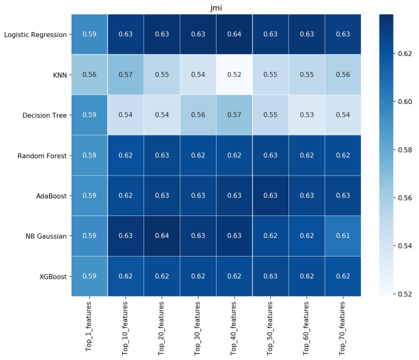

(d) JMI

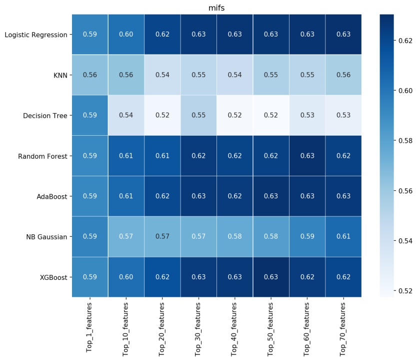

(e) MIFS

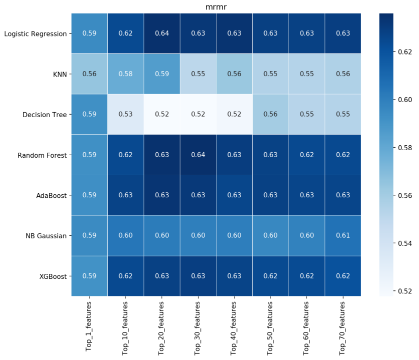

(f) MRMR

Figure 2. Non-Mitigated Feature Selection - Cross-validation.

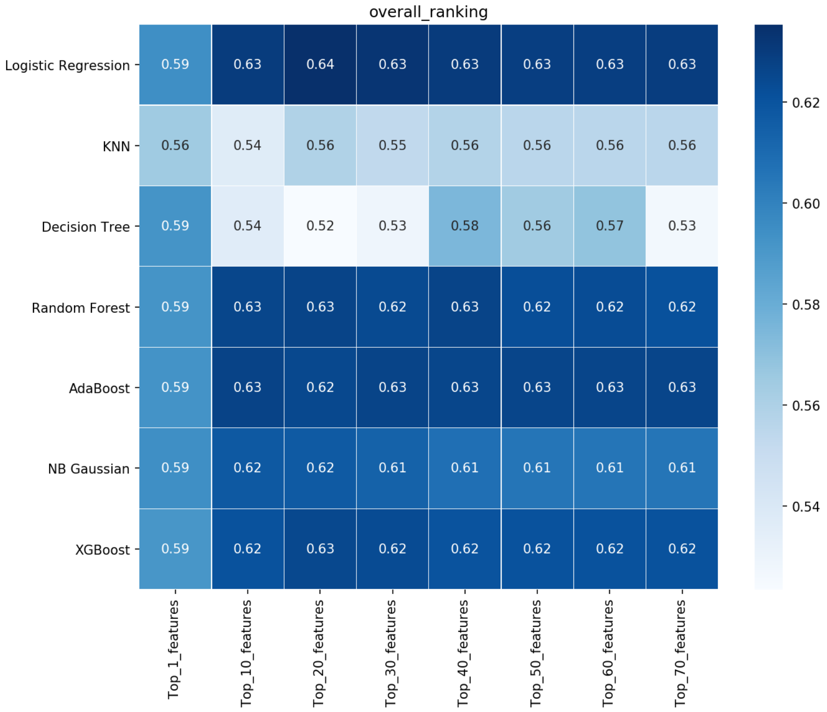

(g) Overall Ranking

Figure 2. Non-Mitigated Feature Selection - Cross-validation.

Model Num	The best models			Accuracy %			F1-score%		
	Model Name	Feature Selec-tion	Top features	Cross validation	Hold out	External	Cross validation	Hold out	External
402	LG	disr	40	0.59	0.54	0.56	0.58	0.53	0.56
303	Adaboost	jmi	60	0.62	0.61	0.53	0.62	0.61	0.56
416	DT	disr	10	0.54	0.54	0.51	0.26	0.25	0.30
398	XGBoost	disr	40	0.58	0.55	0.55	0.58	0.55	0.55
232	KNNC	disr	60	0.73	0.76	0.53	0.76	0.53	0.74
39	NB Gaussain	mim	10	0.56	0.54	0.55	0.45	0.42	0.48
184	Random Forest	icap	20	0.58	0.54	0.57	0.56	0.49	0.58

Table 1. The best generated models out of 448 generated models

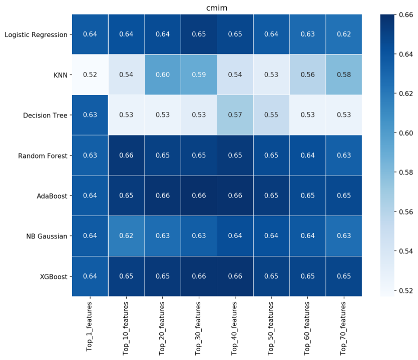

(a) CMIM

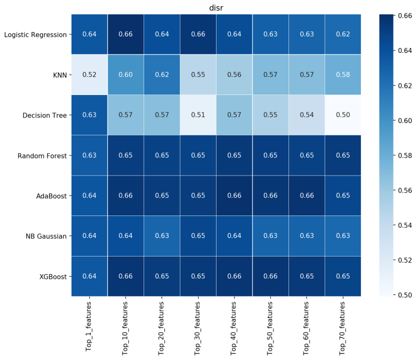

(b) DISR

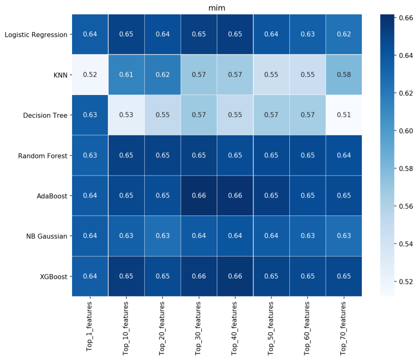

(c) MIM

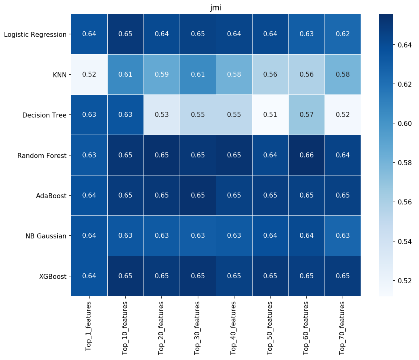

(d) JMI

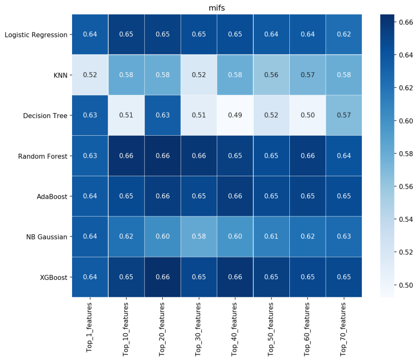

(e) MIFS

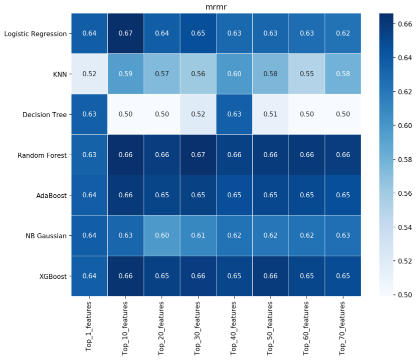

(f) MRMR

Figure 3. Non-Mitigated Feature Selection - hold out.

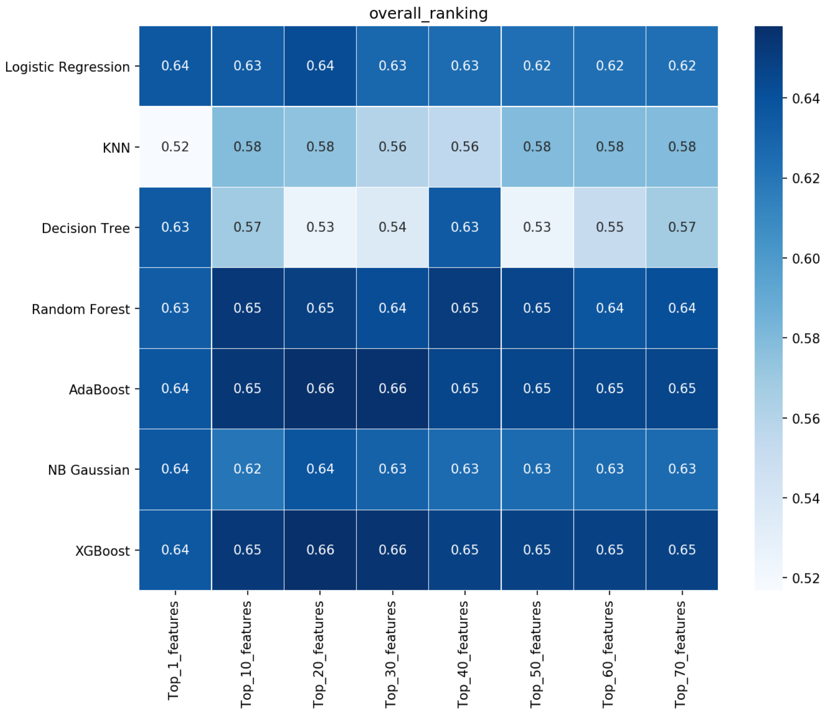

(g) Overall Ranking

Figure 3. Heatmap depicting the predictive performance (AUC for hold out set) for different number of HLA features(in rows) and different classification method in (columns). It can be observed for all feature selection all classifiers show relatively the same predictive performance in many cases. Non-Mitigated Feature Selection -Hold-out.

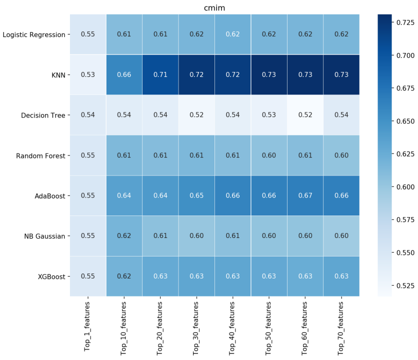

(a) CMIM

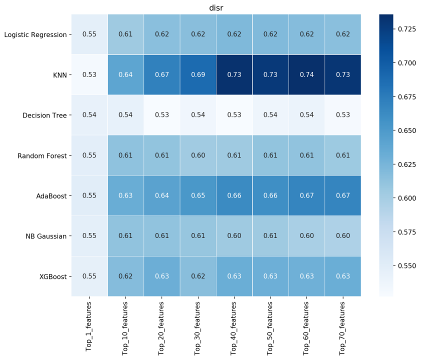

(b) DISR

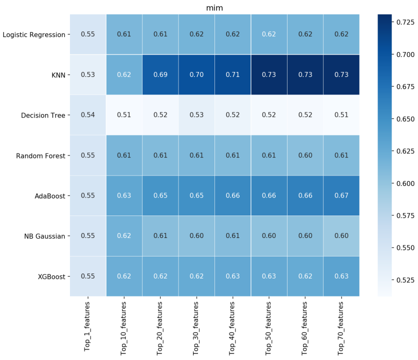

(c) MIM

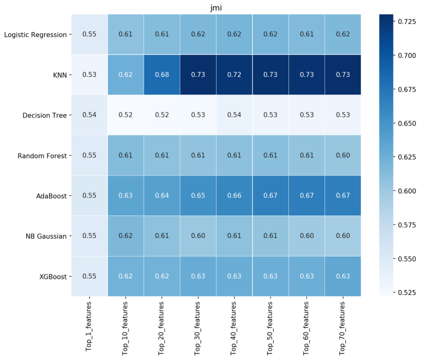

(d) JMI

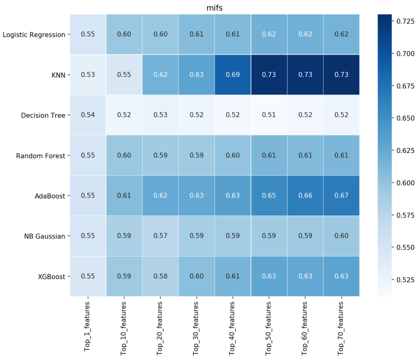

(e) MIFS

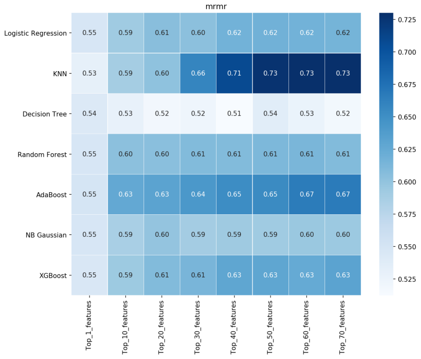

(f) MRMR

Figure 4. Heatmap depicting the predictive performance (AUC average over cross validation) for different number of HLA features(in rows) and different classification method in (columns). It can be observed for all feature selection all classifiers show relatively the same predictive performance in many cases. Mitigated Feature Selection - Cross-validation.

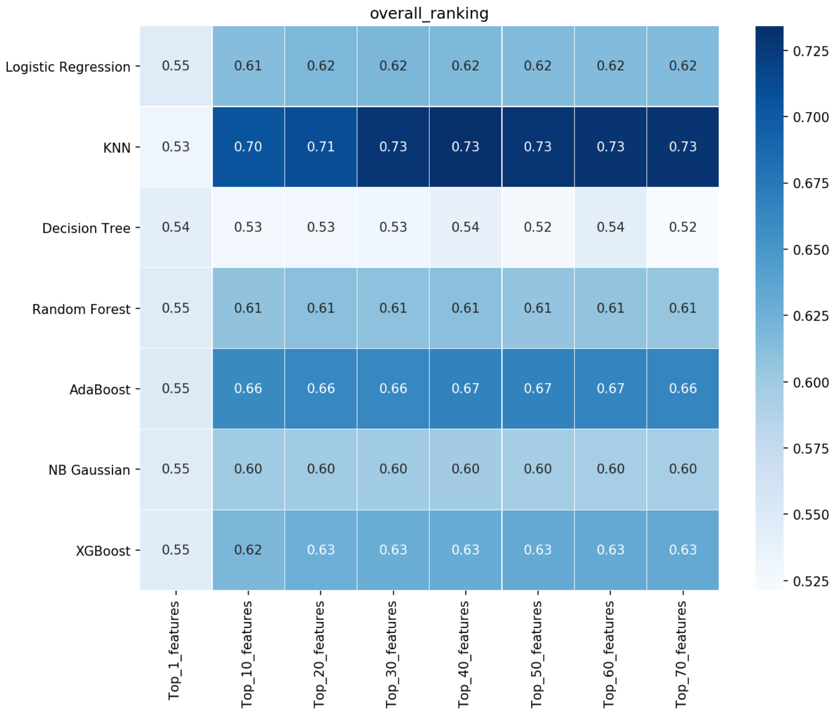

(g) Overall Ranking

Figure 4. Heatmap depicting the predictive performance (AUC average over cross validation) for different number of HLA features(in rows) and different classification method in (columns). It can be observed for all feature selection all classifiers show relatively the same predictive performance in many cases. Mitigated Feature Selection - Cross-validation.

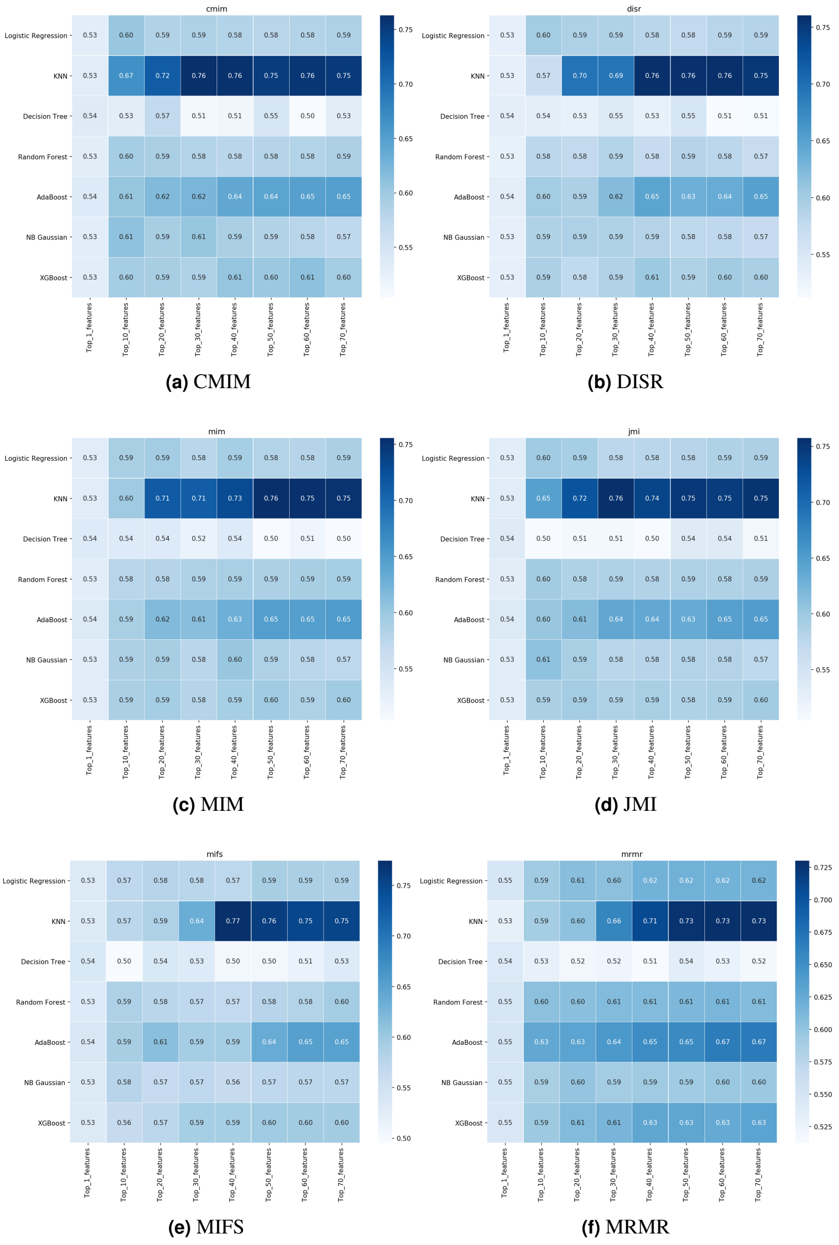

(a) CMIM **(b)** DISR **(c)** MIM **(d)** JMI **(e)** MIFS **(f)** MRMR

Figure 5. Heatmap depicting the predictive performance (AUC hold out) for different number of HLA features(in rows) and different classification method in (columns). It can be observed for all feature selection all classifiers show relatively the same predictive performance in many cases.Mitigated Feature Selection -hold-out.

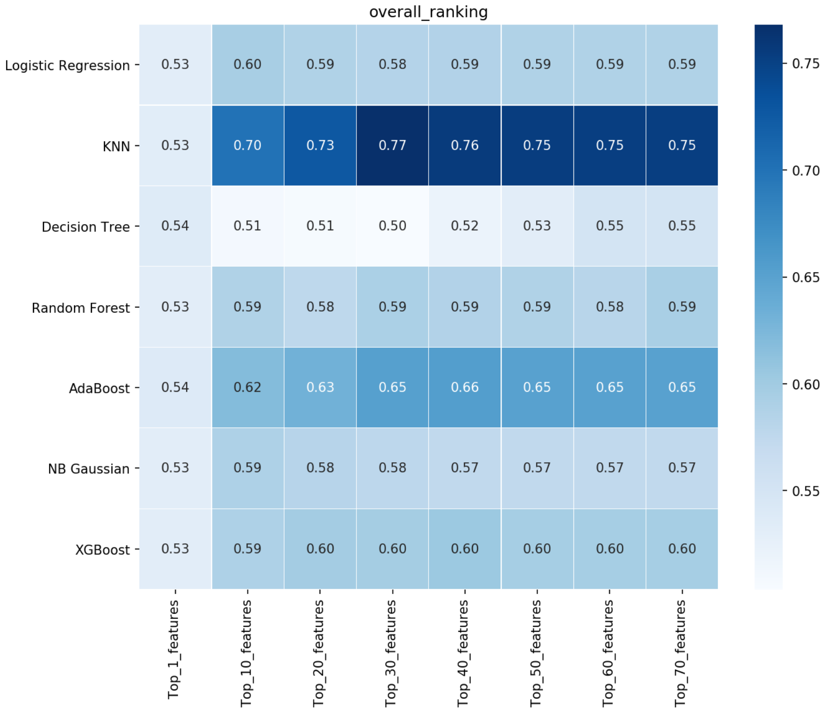

(g) Overall Ranking

Figure 5. Mitigated Feature Selection -hold-out.

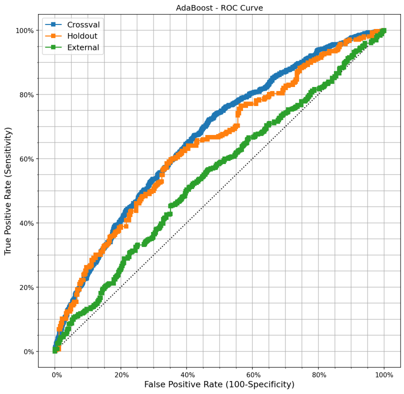

(a) AdaBoost

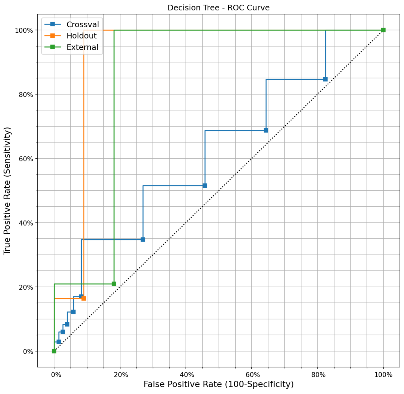

(b) Decision Tree

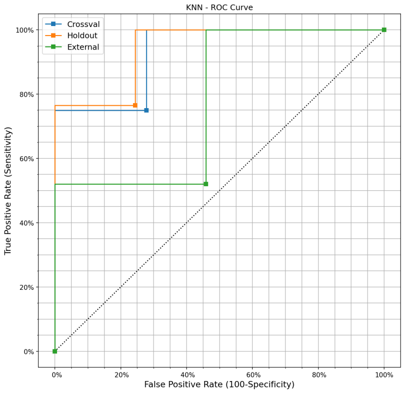

(c) KNN

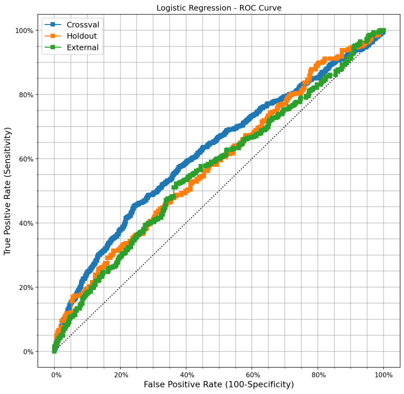

(d) Logistic Regression

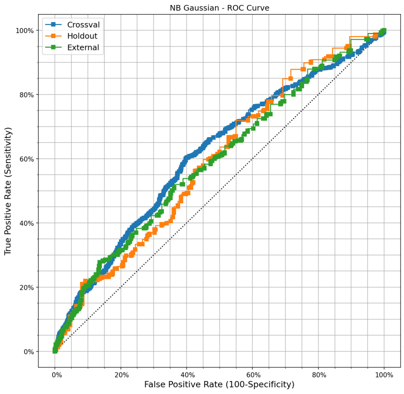

(e) NB Gussian

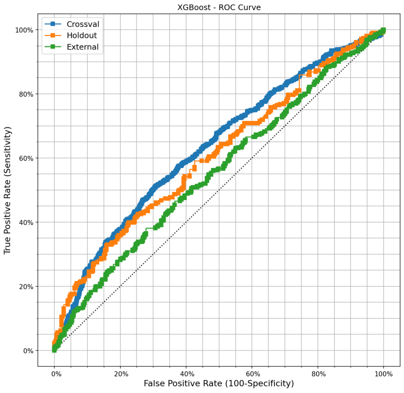

(f) XGBoost

Figure 6. Mitigated - ROC Curve.

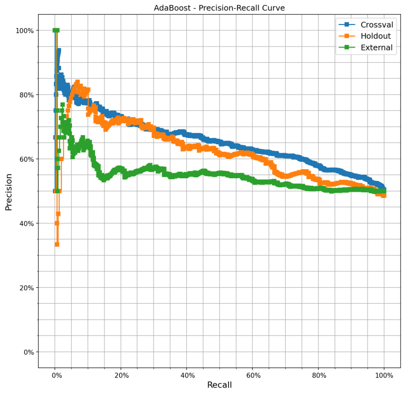

(a) AdaBoost

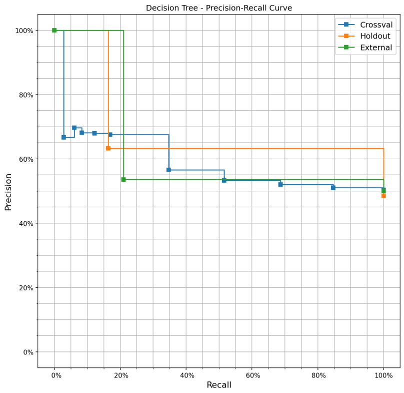

(b) Decision Tree

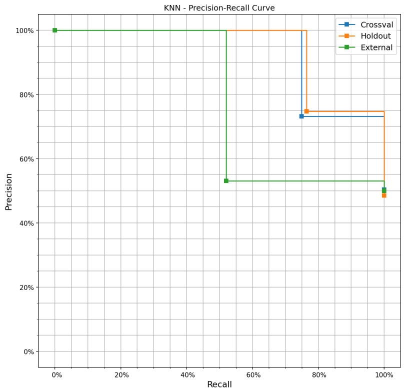

(c) KNN

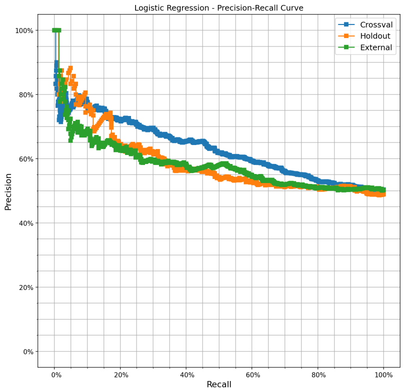

(d) Logistic Regression

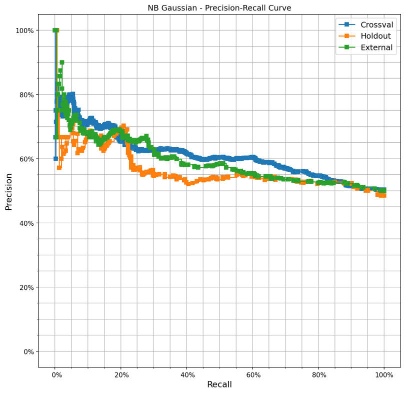

(e) NB Gaussian Forest

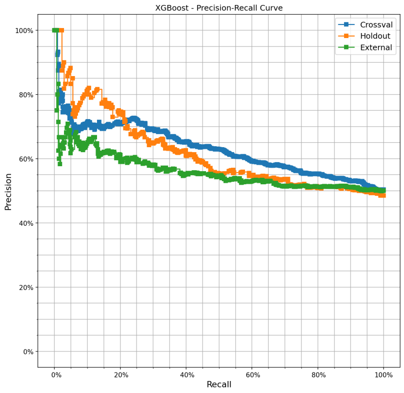

(f) XGBoost

Figure 7. Mitigated - Precision & Recall Plots.

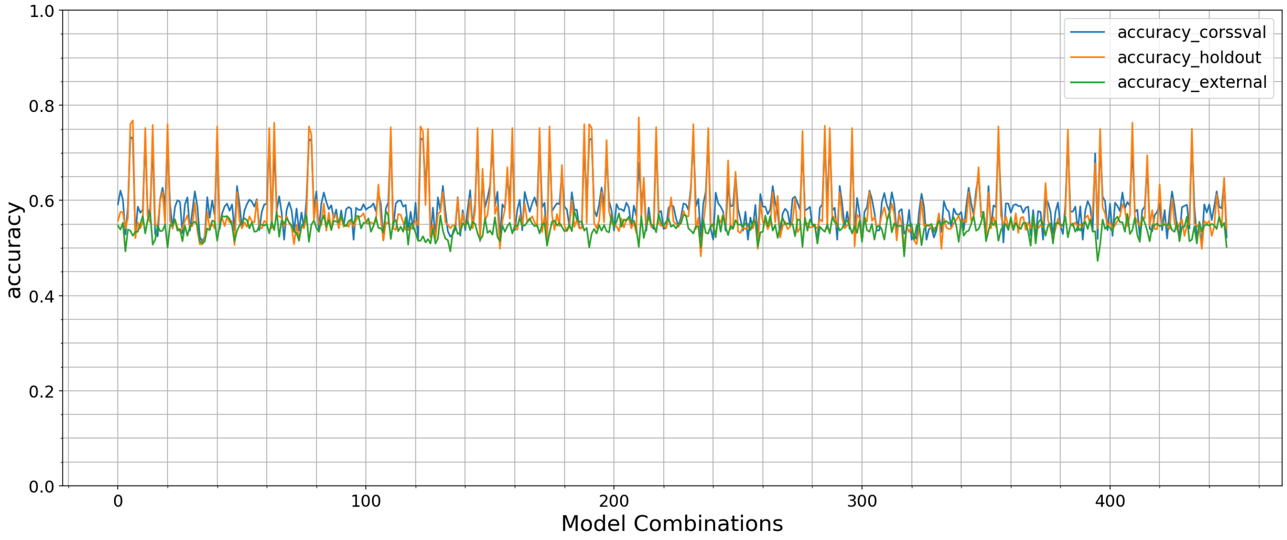

(a) accuracy

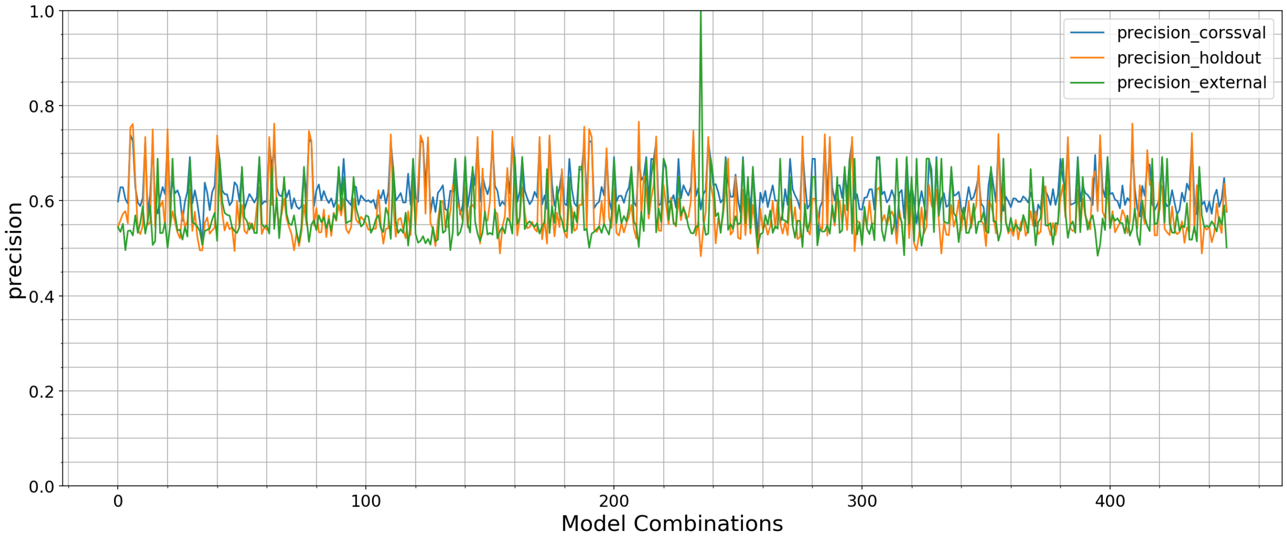

(b) precision

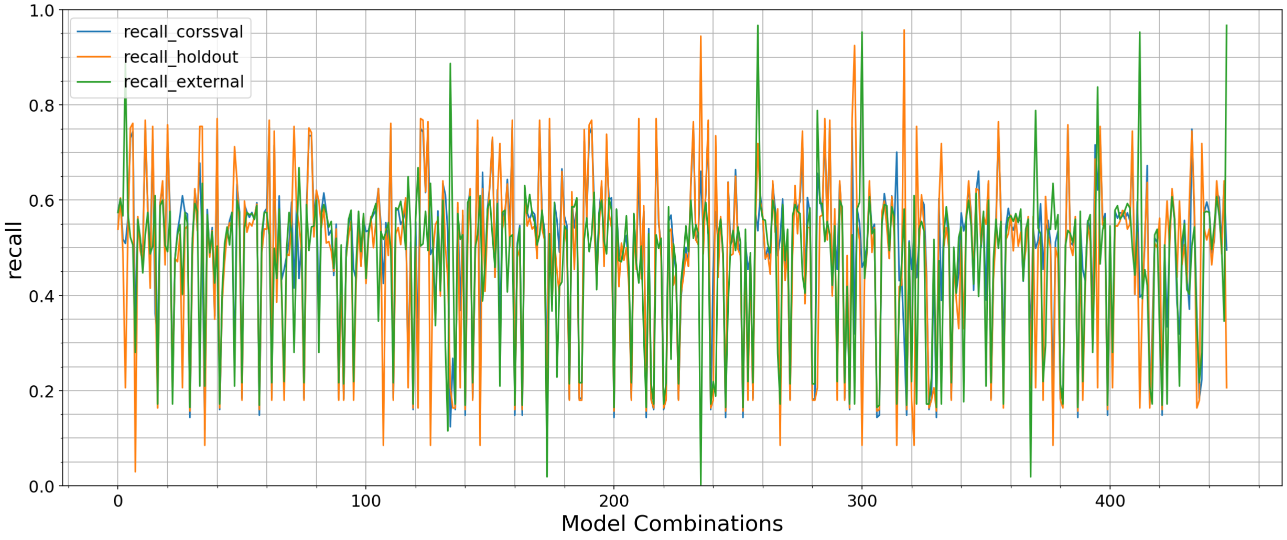

(c) recall

Figure 8. Comparison between accuracy, precision, recall and F1 score for cross validation, hold out and external set for 448 different generated ML models.There are 448 models trained using combination of “number of features”, “Feature Selection”,

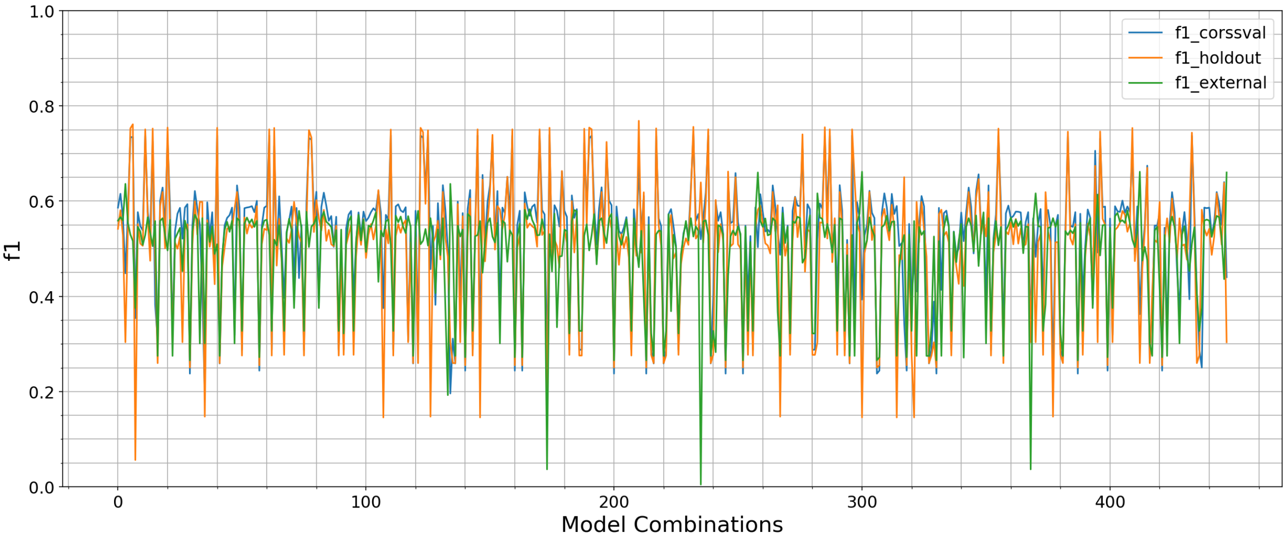

(d) F1-score

Figure 8. Mitigated Feature Selection -hold-out.

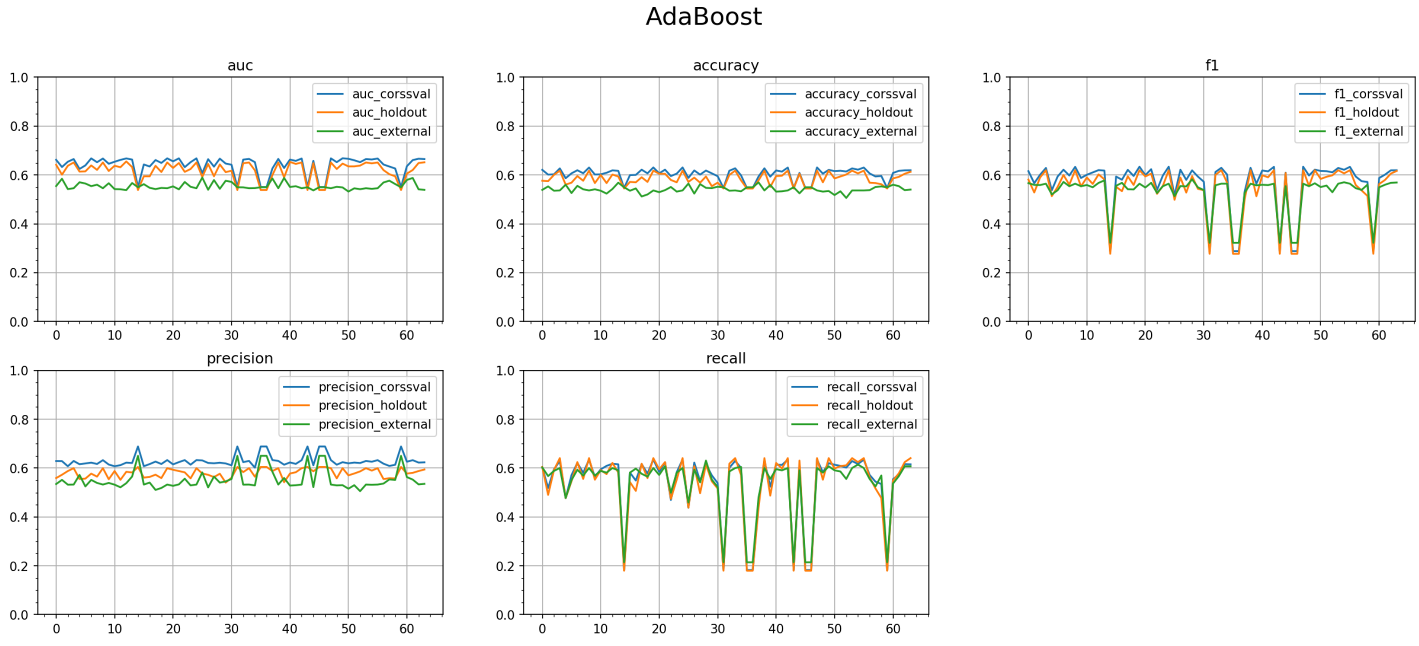

(a) AdaBoost

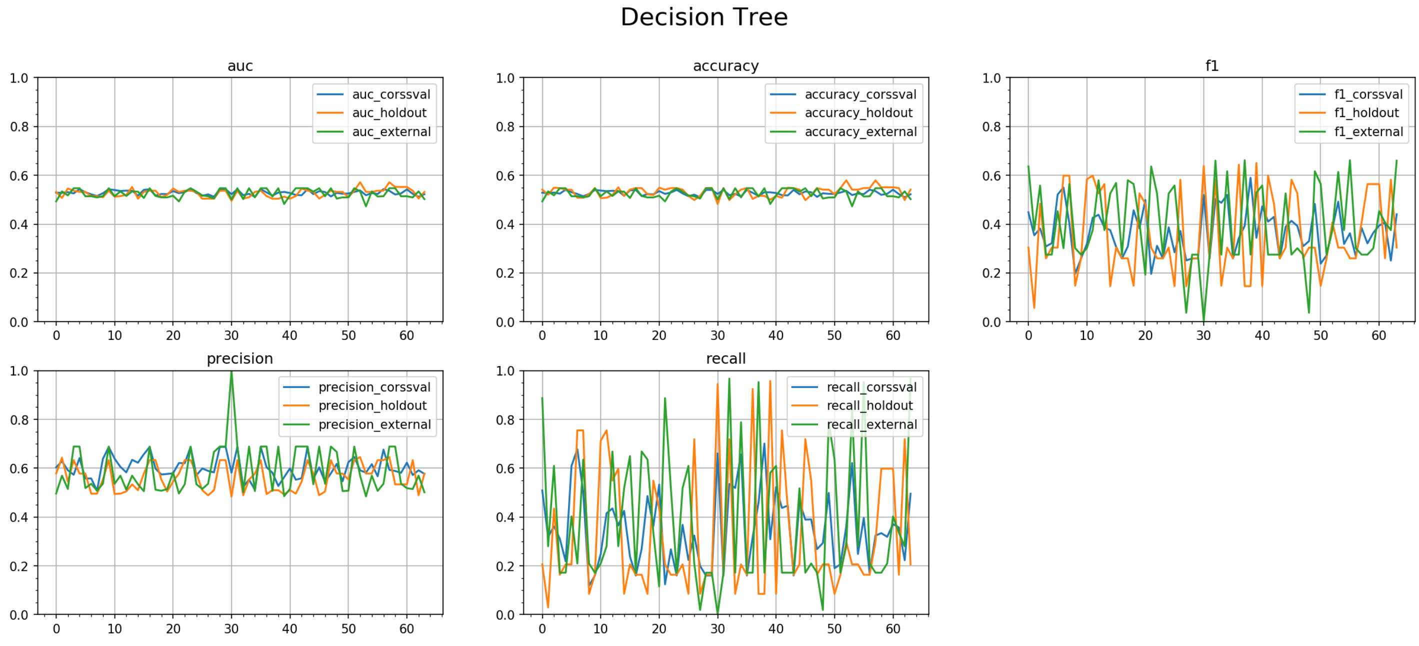

(b) DecisionTree

Figure 9. Accuracy,Precision, Recall and F1 score curve of each models with internal(hold out and cross validation) and external model performance

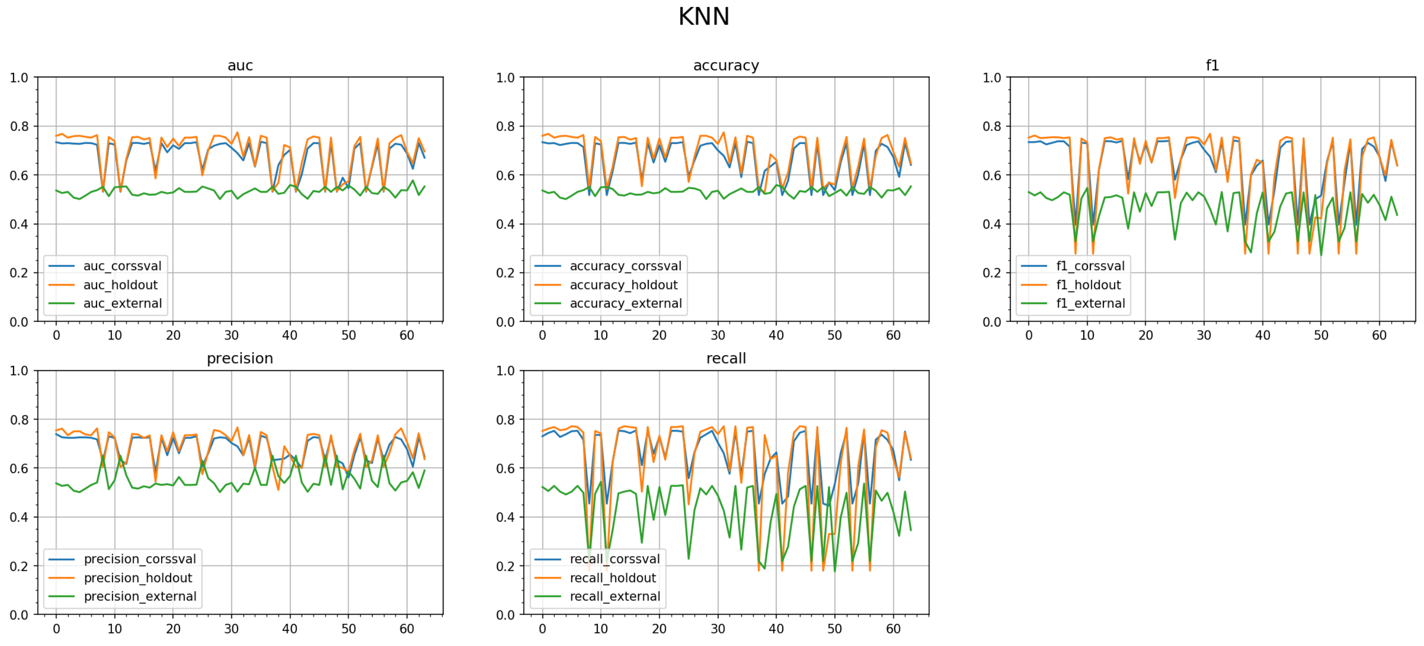

(c) KNNC

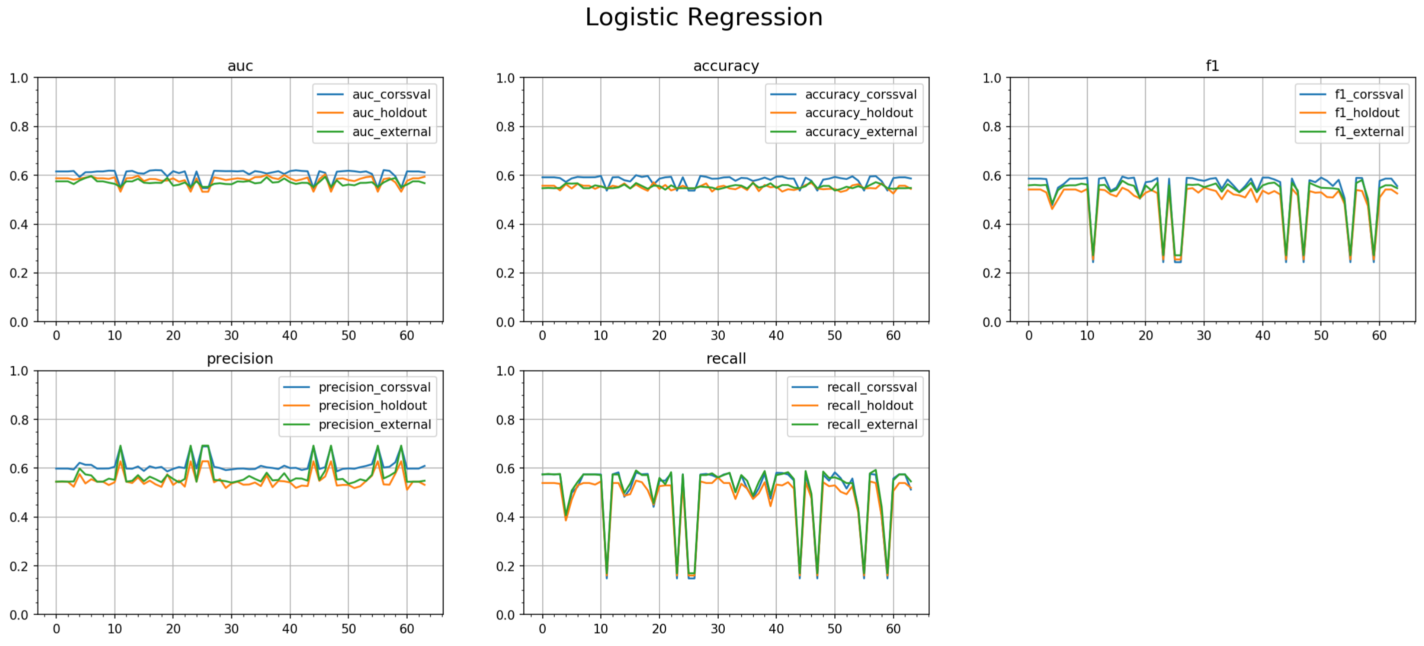

(d) Logistic Regression

Figure 9. Accuracy,Precision, Recall and F1 score curve of the each models with internal(hold out and cross validation) and external model performance

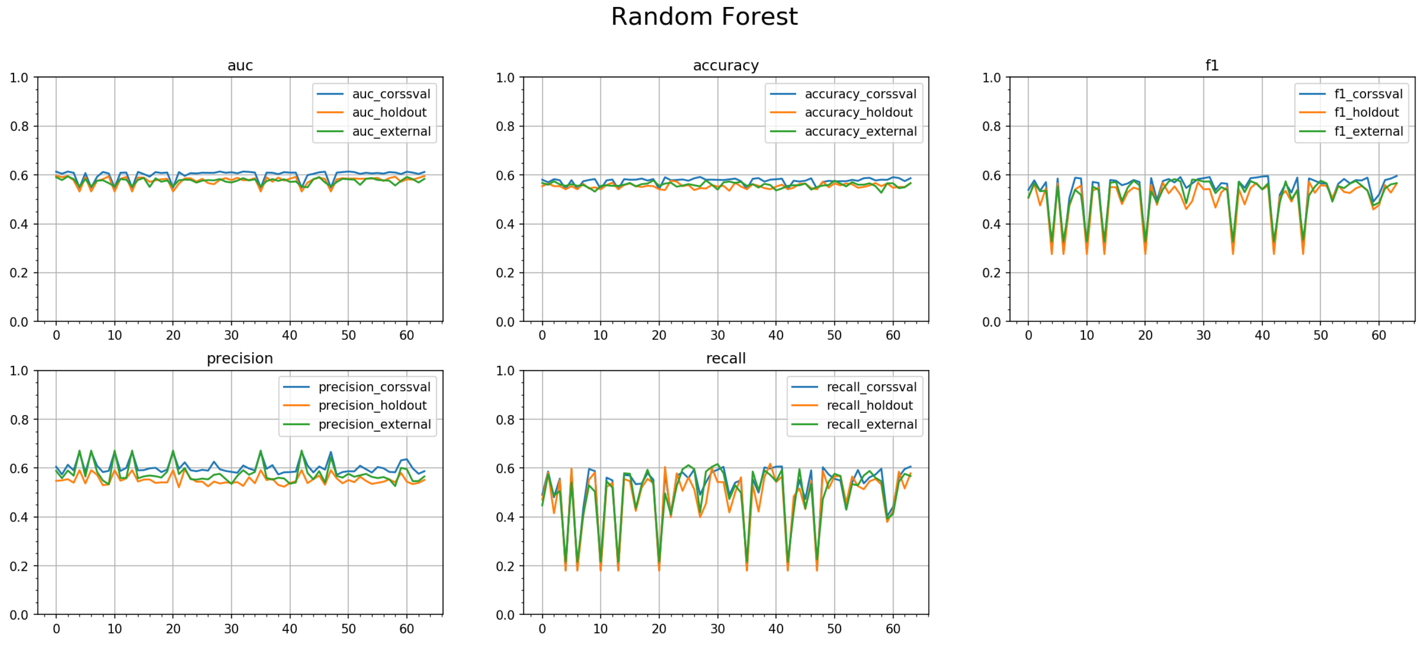

(e) Random Forest

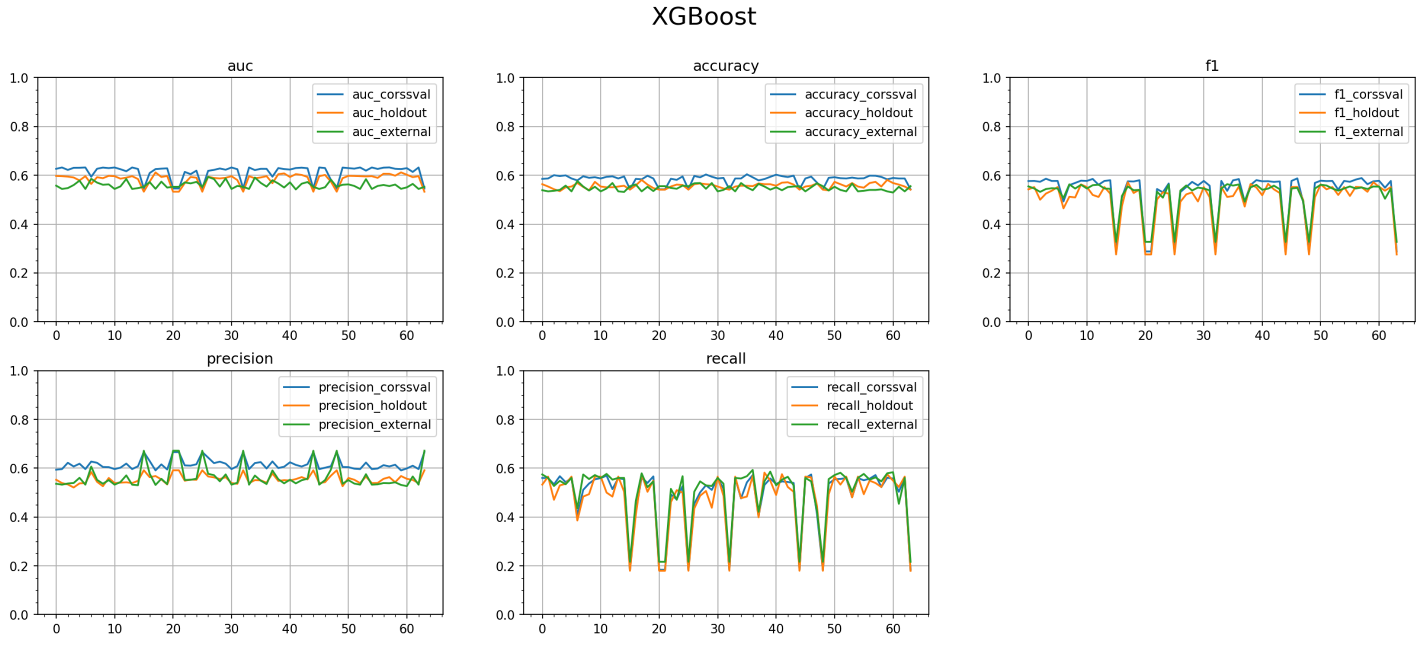

(f) XGBoost

Figure 9. Accuracy,Precision, Recall and F1 score curve of the each models with internal(hold out and cross validation) and external model performance

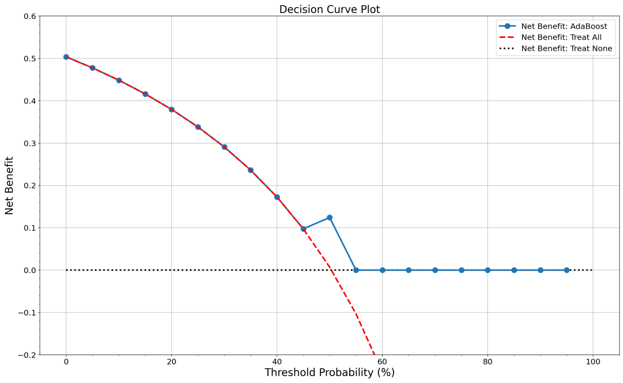

(a) AdaBoost_Cross Validation

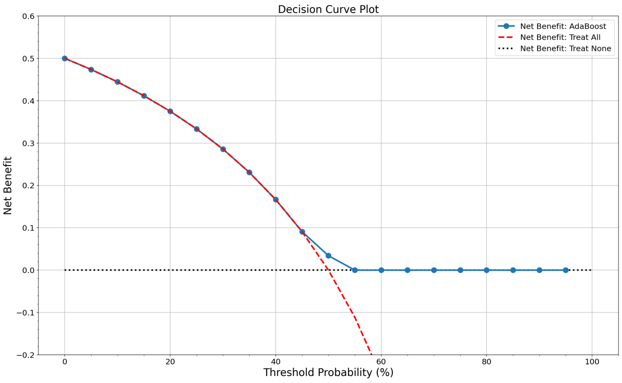

(b) AdaBoost_External Validation

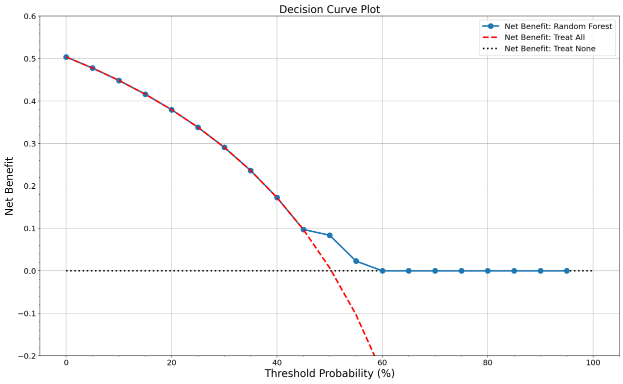

(c) Random Forest_Cross Validation

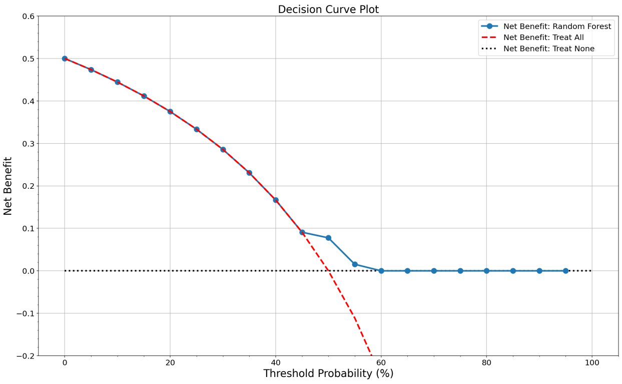

(d) Random Forest_External Validation

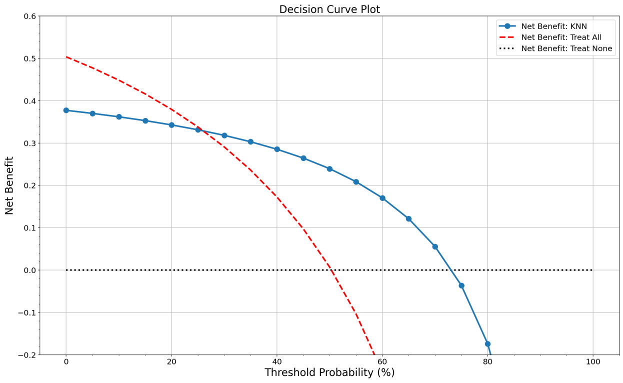

(e) KNN_Cross Validation

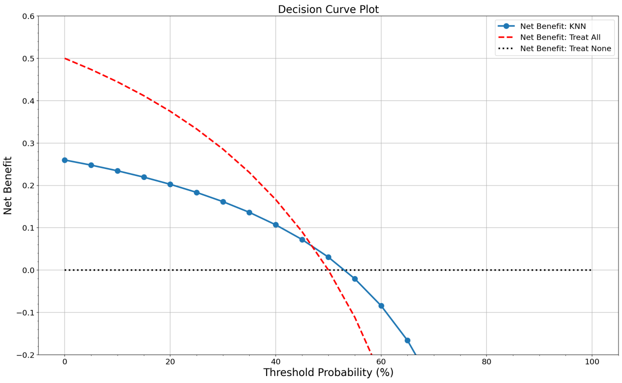

(f) KNN_external

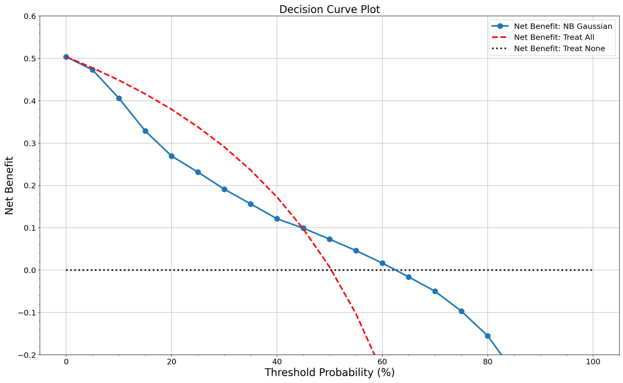

(g) NB_Cross Validation

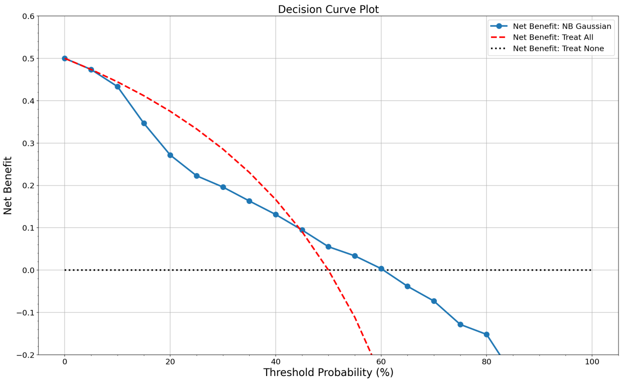

(h) NB_external

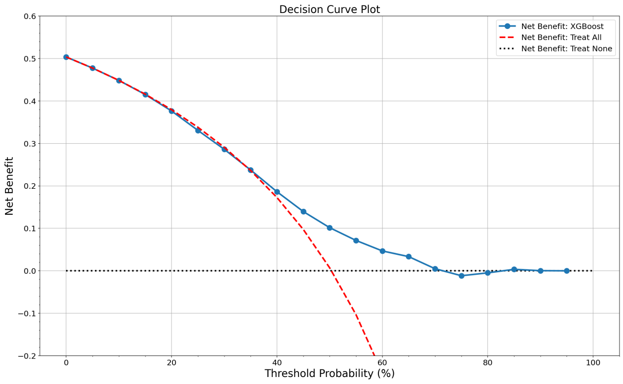

(i) XGBoost_Cross Validation

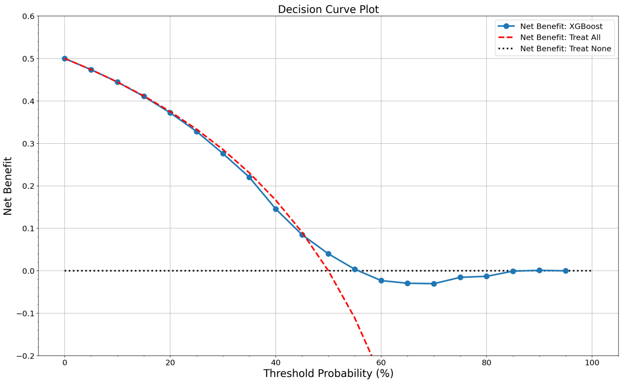

(j) XGBoost_External

Figure 10. Decision curve for each model in cross validation and external set

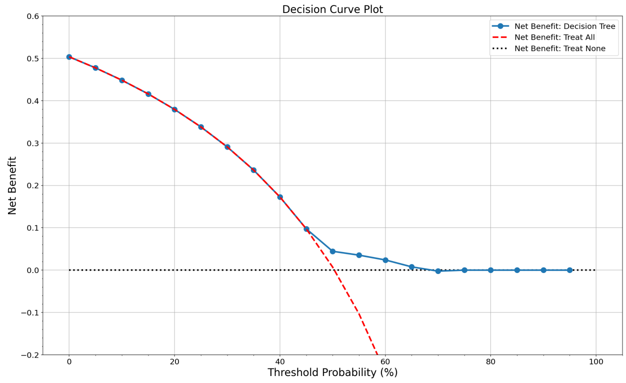

(k) DT_Cross Validation

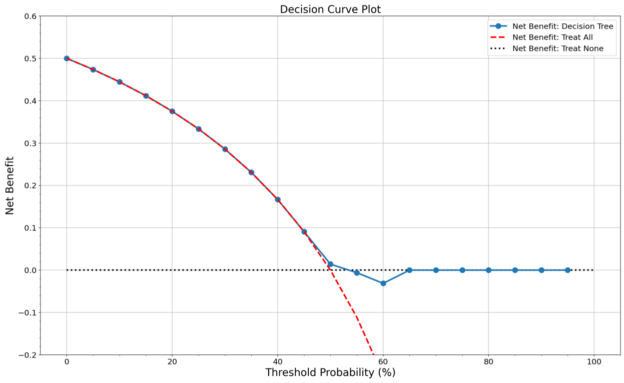

(l) DT_External

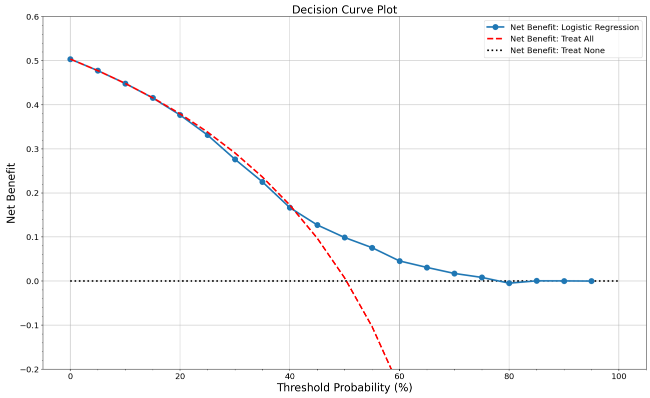

(m) Logistic Regression_Cross Validation

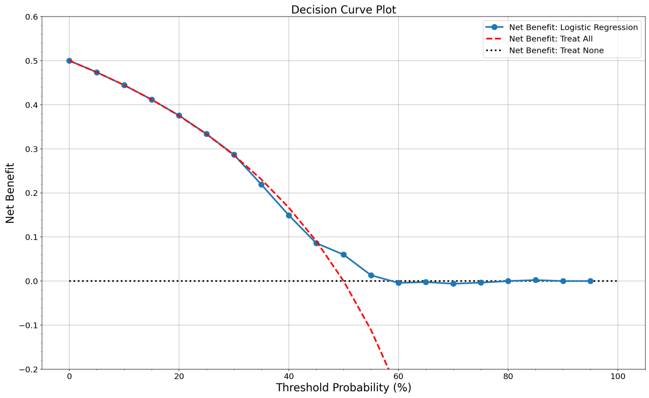

(n) Logistic Regression_External Validation

Figure 10. Decision curve for each model in cross validation and external set
